# Pangenome analyses reveal impact of transposable elements and ploidy on the evolution of potato species

**DOI:** 10.1073/pnas.2211117120

**Published:** 2023-07-24

**Authors:** Ilayda Bozan, Sai Reddy Achakkagari, Noelle L. Anglin, David Ellis, Helen H. Tai, Martina V. Strömvik

**Affiliations:** ^a^Department of Plant Science, McGill University, Sainte-Anne-de-Bellevue, QC H9X 3V9, Canada; ^b^International Potato Center, Lima 15023, Peru; ^c^United States Department of Agriculture - Agricultural Research Service (USDA ARS) Small Grains and Potato Germplasm Research, Aberdeen, ID 1691S 2700W; ^d^Fredericton Research and Development Centre, Agriculture and Agri-Food Canada, Fredericton, NB E3B 4Z7, Canada

**Keywords:** *Petota* pangenome, transposons, adaptation, polyploidy, Solanum

## Abstract

Potato (*Solanum sp*., family Solanaceae) is the most important noncereal food crop globally. It has over 100 wild relatives in the *Solanum* section *Petota*, which features species with both sexual and asexual reproduction and varying ploidy levels. A pangenome of *Solanum* section *Petota* composed of 296 accessions was constructed including diploids and polyploids compared via presence/absence variation (PAV). The *Petota* core (genes shared by at least 97% of the accessions) and shell genomes (shared by 3 to 97%) are enriched in basic molecular and cellular functions, while the cloud genome (genes present in less than 3% of the member accessions) showed enrichment in transposable elements (TEs). Comparison of PAV in domesticated vs. wild accessions was made, and a phylogenetic tree was constructed based on PAVs, grouping accessions into different clades, similar to previous phylogenies produced using DNA markers. A cladewise pangenome approach identified abiotic stress response among the core genes in clade 1+2 and clade 3, and flowering/tuberization among the core genes in clade 4. The TE content differed between the clades, with clade 1+2, which is composed of species from North and Central America with reproductive isolation from species in other clades, having much lower TE content compared to other clades. In contrast, accessions with in vitro propagation history were identified and found to have high levels of TEs. Results indicate a role for TEs in adaptation to new environments, both natural and artificial, for *Solanum* section *Petota*.

## Significance

The *Solanum* section *Petota* includes potato cultivars, landraces, and wild species. The latter are a source of genetic diversity for biotic and abiotic stress resistance, nutritional and adaptive traits—critical for adaptation to climate change, increased sustainability of crop production, and improvement of nutritional quality for health and food security. This study compares diploid and polyploid species across section *Petota* by looking at present and absent genes at a pangenome scale, well reflecting the taxonomic tree. Reproductively isolated clades show differences in transposable elements (TEs), providing evidence for a role of TEs in plant speciation and a new perspective on the complexity of potato taxonomy.

Potato (*Solanum tuberosum* L.) is the third most important crop and the most important noncereal crop for food security in the world (1). It is part of the *Solanum* section *Petota*, which has over 100 species adapted to a variety of seasonality and precipitation (2, 3). The highest diversity of wild species of *Solanum* section *Petota* is found in high-elevation, cold-adapted montane habitats in central Mexico and central Andes (2–4), and cultivated species and their landraces are derived from western Venezuela to southern Chile, with the center of origin at Lake Titicaca, which straddles Peru and Bolivia (5).

Domestication occurred approximately 10,000 y ago in the Andean highlands (now southern Peru), where different potato landraces are still grown at high elevation (3,000 to 4,000 m) (5, 6). Domestication resulted from human and natural selection for larger tubers, lower glycoalkaloid content, and the selection of attractive tuber shapes and colors. Many potatoes further hybridized or underwent ploidy changes, leading to a vast genepool (7) consisting of more than 4,500 native landrace potatoes (referred to as “papas nativas” in Peru) (8).

Ploidy levels are a major breeding barrier in many crops while introgressing genetic material from wild species. The basic (haploid) chromosome number for potatoes is 12, though ploidy ranges from diploid to hexaploid with the majority of species classified as diploids (9). The landraces range from diploid to pentaploid. Polyploidization (and then speciation) increases with harsher environments, especially cold and dry, and with biotic stress (10). Most of the modern commercially grown cultivated potatoes, especially in North America and Europe, are autotetraploid (2n=4×=48), though both diploids and polyploids are used in breeding to introgress specific traits. Occasionally, the diploid cultivated and wild potato relatives produce 2n gametes allowing them to hybridize with tetraploids. Triploid species are thought to result from interploidy hybridization between n and 2n gametes (11). However, the Andean cultivated tetraploids (*S. tuberosum* group Andigena; 2n=4×=48) are the result of autopolyploidization of diploids (11).

Nowhere is biodiversity used more than in farming systems throughout South America to get the most out of the cultivation resources for food security. Cultivated and wild potatoes are of great global interest, and many potato genomes have been sequenced and assembled (12–23). Yet, because *Solanum* section *Petota* is complex with species showing variable ploidy levels, both asexual and sexual reproduction, self-incompatibility, prezygotic and postzygotic reproductive barriers (24) and because polyploid genome sequences are difficult to resolve (25), larger potato genome studies to date have either used only diploids (23) or tetraploids (21), and no pangenome assembly is available that integrates and synthesizes the vast genetic diversity and different ploidy levels of this section. A pangenome can answer larger questions of relatedness and adaptation and provide greater knowledge of the diversity of a grouping of interest.

Here, we present a *Petota* pangenome based on sequences from 296 diploid and polyploid accessions of different species, landraces, cultivars, and commercial varieties, representing the most taxa-inclusive potato pangenome to date. It was constructed by using the *S. tuberosum* group Tuberosum RH89-039-16 v3 (hereafter referred to as RH) reference genome (19), to which ~7.3 Gbp of additional sequences and 56,843 additional genes were added for a total of 132,355 pangenes. Presence/absence variation (PAV) data were used to investigate the core, shell, and cloud genome of the *Petota* pangenome along with cladistic differences in the member accessions. Transposable elements (TEs) were found to vary between clades and were particularly prevalent in in vitro propagated accessions. A PAV-based clustering was used to construct a phylogenetic tree, that supports previous taxonomic groupings and reveals gene functions involved in adaptation to environmental and climate variation, cultivation practices, in addition to providing insights into TE and speciation in section *Petota*.

## Results

### The Pangenome of *Solanum* Section *Petota*.

A total of 296 diverse potato accessions were used to construct a superpangenome for the *Solanum* section *Petota* to harness the vast information in current genome sequence data and reveal variation not present in existing reference genomes. These accessions represent a broad range of wild species, landraces, and cultivars with different ploidy levels (i.e., 2×, 3×, 4×, and 5×), and varying geographical origins. Publicly available genome assemblies of 33 accessions were used directly in the pangenome construction, while the remaining accessions were assembled de novo to generate a total of 154 Gbp of contigs (*SI Appendix*, Table S1 and Fig. S1). The short-read assemblies have N50 values in the range of 1,393 to 16,809 bp, whereas the long-read assemblies have a better contiguity and completeness (*SI Appendix*, Fig. S2). These assemblies along with publicly available genome assemblies were aligned to the reference genome (RH) to get nonreference sequences. These nonreference sequences were subjected to multiple rounds of redundancy removal to obtain a final set of nonredundant nonreference sequences. The novel unaligned sequences added to the reference consisted of 5,146,040 contigs that have <90% identity to the reference with at least 500 bp length totaling ~7.3 Gbp in size, of which ~5.9 Gbp (79.5%) is composed of repetitive elements. The major part of the nonreference genome is composed of sequences from clade 1+2 and clade 3 wild species (*SI Appendix*, Fig. S3). All the nonreference sequences were added to the reference genome to make the section *Petota* pangenome. The pangenome consists of 12 chromosomes (haplotype-resolved, 24 haplotypes) plus 5,148,884 contigs with a total size of ~9.05 Gbp. There are 37,083 contigs in the pangenome with at least 10 kbp and 176,938 contigs with at least 5 kbp in size. There are 2,957,143 (~57.4%) contigs with >90% of repetitive sequences. The pangenome consists of 100% of the BUSCO orthologs from the *Viridiplantae* dataset, as well as more than 99% of the orthologs from the *Embryophyta* and *Solanales* datasets (*SI Appendix*, Fig. S4). Annotation of the nonreference sequences generated a total of 56,843 gene models representing 54,135 nonredundant genes. Among the nonreference genes, a total of 42,779 genes were assigned functions and/or Pfam domains through functional annotations. These nonreference genes were added to the RH reference annotations to generate a total of 132,355 genes in the pangenome.

### PAV Variation in Protein-Coding Genes.

**Fig. 1.**
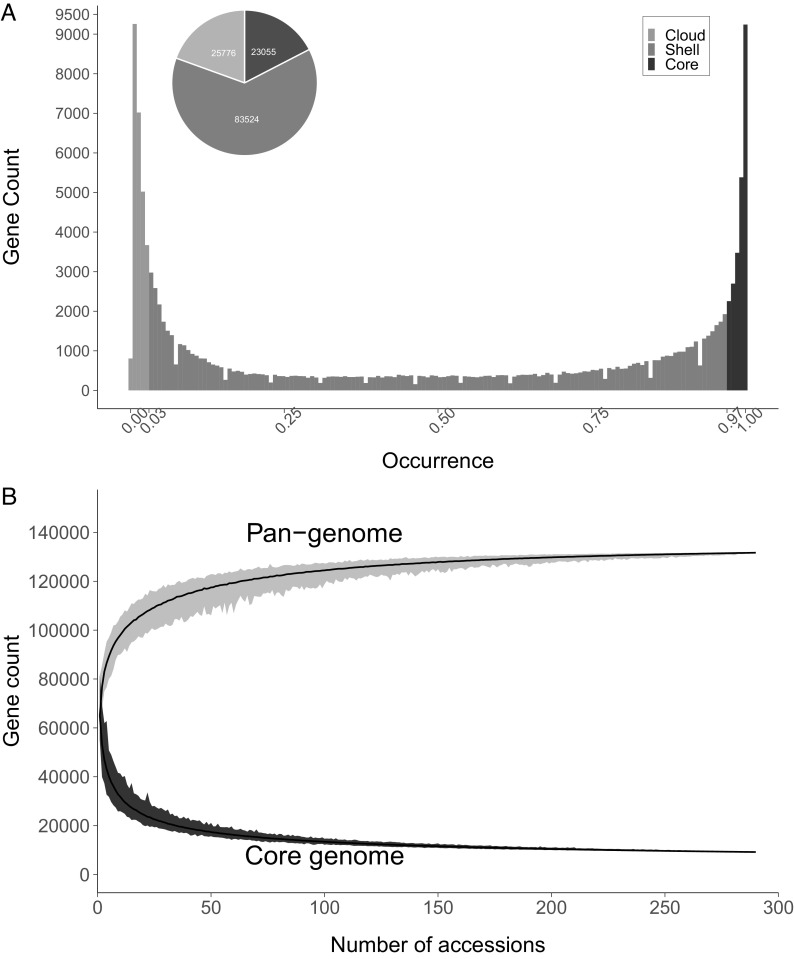
The *Solanum* section *Petota* pangenome. (*A*) Histogram of the core, shell, and cloud genes in the *Solanum* section *Petota* pangenome with a pie chart showing the numbers of genes found in each category. (*B*) The rarefaction curves show an increase in the pangenome size and decrease in the core genome size as more accessions are added. The pangenome reaches a plateau around 80 accessions.

PAV can identify gene diversity and variation among individual accessions (26). Several pangenomic studies have successfully used PAV data to understand gene content variation and its effects between populations (26–29). Effective PAV calling can be performed with low-depth sequences and a map-to-pan approach, which has been shown to have minimal effects on the amount of sequence used in PAV calling (30). We performed the PAV analysis in this study to include accessions with low read depth. The PAV analysis showed that the number of genes in each accession varied from the lowest in the diploid wild *S. polyadenium* (PI 347770) with 54,867 genes to the highest in a triploid and pentaploid, *Solanum juzepczukii* (CIP 706050) and *Solanum curtilobum* (CIP 702937), with 81,418 and 81,246 genes, respectively. The presence frequencies were calculated for each gene, which were categorized into three groups (core, shell, and cloud) based on their overall frequencies (Fig. 1). There are 23,055 core genes in most of the accessions (>97% of the accessions) (Fig. 1*A*). Similarly, there are 25,776 cloud and 83,524 shell genes present in <3% and 3 to 97% of the accessions, respectively (Fig. 1*A*). A greater number of variable genes and a very low number of core genes were observed in the *Petota* pangenome, demonstrating the variability existing in *Solanum*, as was also previously shown in a pantranscriptome study where a total of 96,886 nonredundant representative transcripts was reported, and the number of cultivar-specific genes was larger than the number of core genes in the assembly of only four potato genotypes—*S. tuberosum* group *phureja* (DM) and three tetraploid group tuberosum (cv. Désirée, cv. Rywal, and breeding clone PW363) (31).

This pangenome consists of many more species (especially wild species) than any previous plant pangenome (23, 26–28, 32) and so could be seen as a superpangenome or supergenome. As more genomes were added, the number of core genes decreased, and the overall pangenome increased rapidly, until a plateau was reached after adding about 80 genomes (Fig. 1*B*). The core genome is functionally enriched in fundamental molecular and cellular processes with cellular protein modification processes (GO:0006464) and phosphorus metabolic processes (GO:0006793) showing the highest numbers of genes with low p-values (*SI Appendix*, Fig. S5). While the shell genome is enriched in regulation of RNA biosynthesis (GO:2001141), response to stress (GO:0006950), and translation (GO:0006412) with functions in pathogen response, response to nutrients and development, as well as in triterpenoid biosynthesis (GO:0016104) (genes involved in synthesis of glycoalkaloids—the typical defense metabolites of *Solanum* plants), the cloud genome is notably enriched in response to water (GO:0009415), DNA-mediated transposition (GO:0006313), embryo development (GO:0009793), DNA integration (GO:0015074), and regulation of RNA biosynthesis (GO:2001141), with functions related to TE (transposition) functions, regulation of gene expression, stress responses, and photosynthesis.

**Fig. 2.**
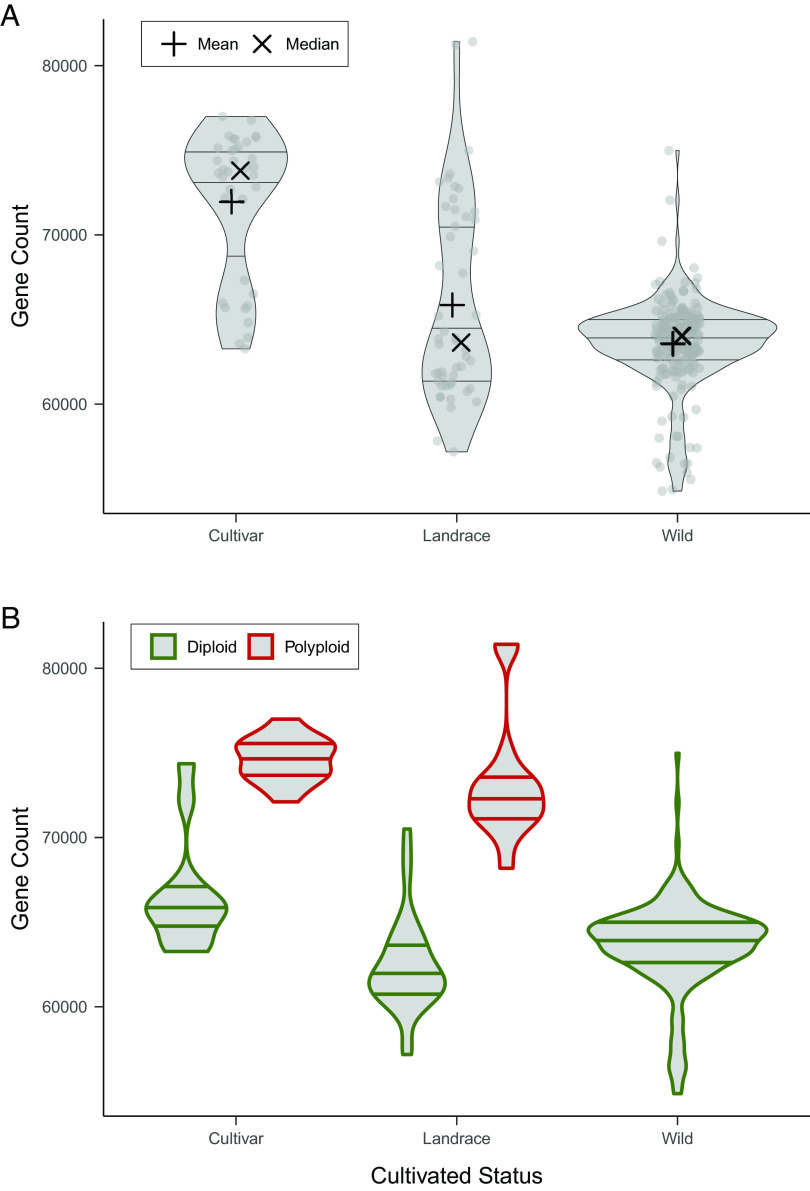
Gene content of the accessions included in the *Solanum* section *Petota* pangenome based on PAV. (*A*) Distribution of the genes in wild species, landrace, and cultivar *Petota* accessions. Lines in the violin plot show the location of the upper quartile, the median, and the lower quartile. The “+” sign is the weighted mean, and the “X” is the median. (*B*) Distribution of the genes in the wild species, landrace, and cultivar accessions with different ploidy levels.

The mean gene count of wild species is considerably lower compared to the landraces and cultivars, with 63,567, 65,856, and 71,950, respectively (Fig. 2*A*). Moreover, the polyploids have more genes compared to the diploids, and diploid cultivars have more genes than the diploid wild species (Fig. 2*B* and *SI Appendix*, Fig. S6), showing that the increase in gene content in potatoes is mainly driven by ploidy, but domestication and breeding also play a role. Curiously, the diploid outliers—the ones with much higher gene content in the wild group—were all conserved for many years in tissue culture, compared to the rest of the wild diploid species that were derived from single-seed descent (Fig. 2*A*).

To identify signals of selection in PAVs for landraces and cultivars, the frequencies of genes between the wild species, landraces, and cultivars were compared. The genes with significant PAV differences were identified, and genes with higher frequencies in landraces or cultivars compared to the wild species were categorized as “favorable” genes, and the ones with lower frequencies than in the wild species were categorized as “unfavorable” genes (27, 33). A total of 8,684 favorable genes and 4,814 unfavorable genes were identified among the cultivars and landraces. Moreover, there were 3,617 landrace-specific favorable genes (where landrace presence frequency > cultivar presence frequency) and 4,717 cultivar-specific favorable genes (cultivar frequency > landrace presence frequency) (*SI Appendix*, Fig. S7). The enrichment analysis indicated functions related to stress response, embryo development ending in seed dormancy, root development, cellular glucose homeostasis, and photosynthesis in cultivar-favorable genes. Mismatch repair, photosynthetic electron transport chain, regulation of translational fidelity, and autophagy of peroxisomes were enriched among landrace-favorable genes (*SI Appendix*, Fig. S8). These results suggest that domestication involved genome variation from gene duplication and loss processes. The functional analysis of the genes with PAV suggests improvement in photosynthesis, modification in metabolism, and development in landraces and cultivars compared to wild species.

### Clustering the Accessions Based on Gene Content.

**Fig. 3.**
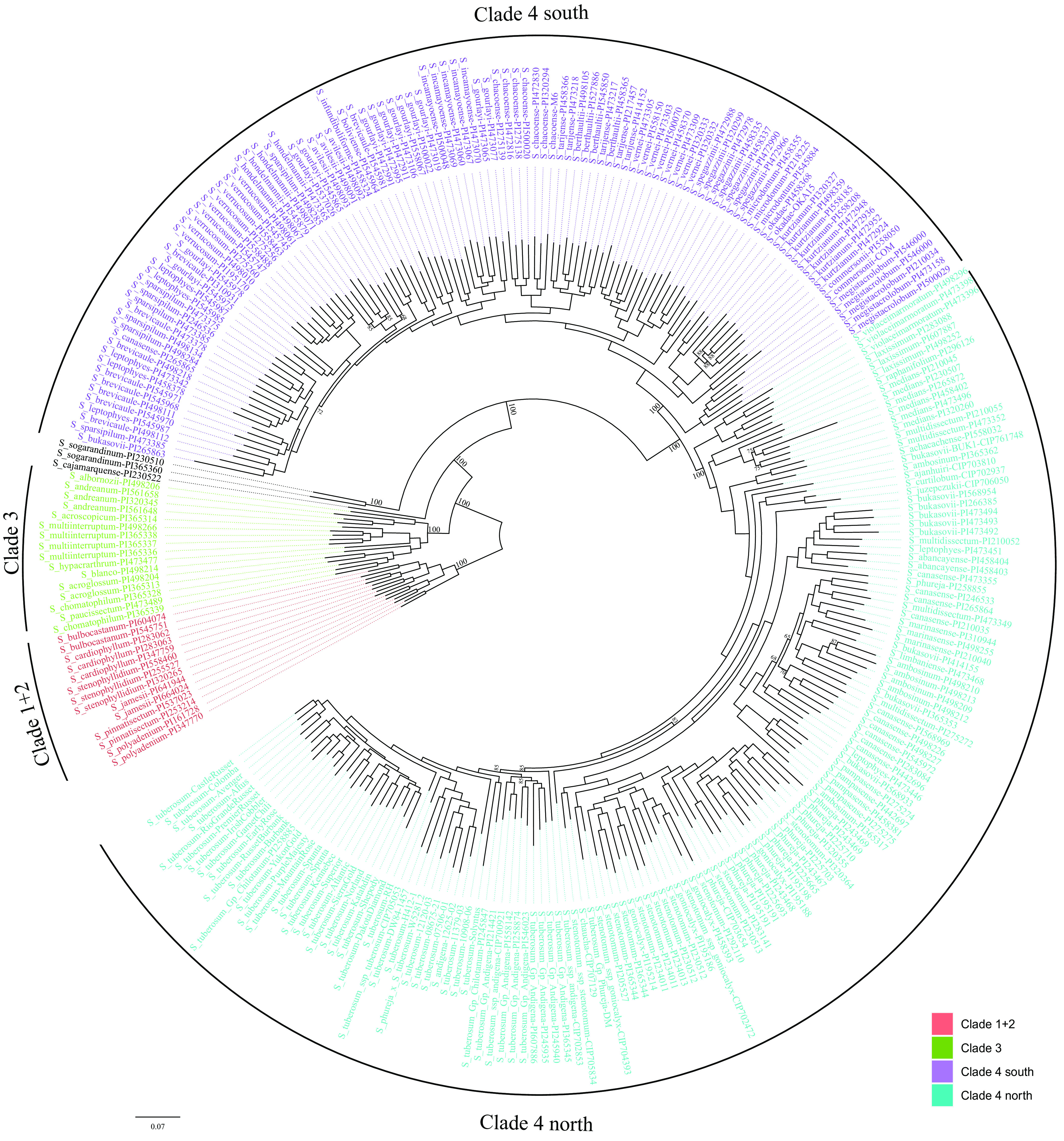
A maximum likelihood phylogenetic tree constructed using PAV data for the *Solanum* section *Petota* pangenome. The accessions are grouped into three major clades with strong bootstrap support (100): clade 1+2, clade 3, and clade 4. Clade 4 is further separated into clade 4 south and clade 4 north. Bootstrap values over 90, except those at the main nodes, were removed to make the figure clearer.

The PAV data were used for constructing a maximum likelihood phylogenetic tree (Fig. 3) to compare the PAV-based groupings with the existing clade concepts in potato. This phylogenetic tree is in agreement with previous phylogenetic studies (2, 14, 15, 23, 34). A clear separation of different clades is observed in this classification, as also seen in ref. 15. Accessions in the PAV-based phylogeny followed previously classified clades 1+2, 3, and 4. Clade 4 was subdivided as clade 4 north and south. All of the accessions previously classified as clade 3 species grouped together, except for the ambiguous species, *Solanum cajamarquense* and *Solanum sogarandinum*. The latter has previously been considered a clade 4 species (2) or labeled as clade 3 (15) as summarized in Solanaceae Source (35); however, the present tree placed it outside both clade 3 and clade 4 in a separate group of its own, similar to what was reported in a previous study (23). This particular accession of *S. cajamarquense* (PI 230522) is labeled as “hybrid seed” and grouped with clade 4 south in the plastome data (34), whereas the nuclear data recorded different placements for this accession by Singular Value Decomposition (SVD) quartets and Maximum Likelihood (ML) analyses (15).

The clade 4 species further separated as clade 4 south and clade 4 north groups as also shown in other studies (2, 15, 34). The clade 4 south group consists of species from the southern region including Bolivia and Argentina, as well as the *Solanum verrucosum* species from Mexico (*SI Appendix*, Fig. S9). The northern group consists of wild species that were from Peru, as well as landraces and cultivars. The groupings observed in the northern group are very interesting. The diploid landraces (*Solanum phureja, Solanum stenotomum,* and *Solanum goniocalyx*) are grouped together and share similar gene loss/gains with the *Solanum candolleanum* species (*Solanum bukasovii, Solanum multidissectum, Solanum abancayense, Solanum canasense, Solanum marinasense, Solanum ambosinum,* and *Solanum pampasense*). All the Andigena and Tuberosum accessions are grouped closely with each other and with the bitter potatoes (*S. curtilobum*, *Solanum ajanhuiri*, and *S. juzepczukii*).

**Fig. 4.**
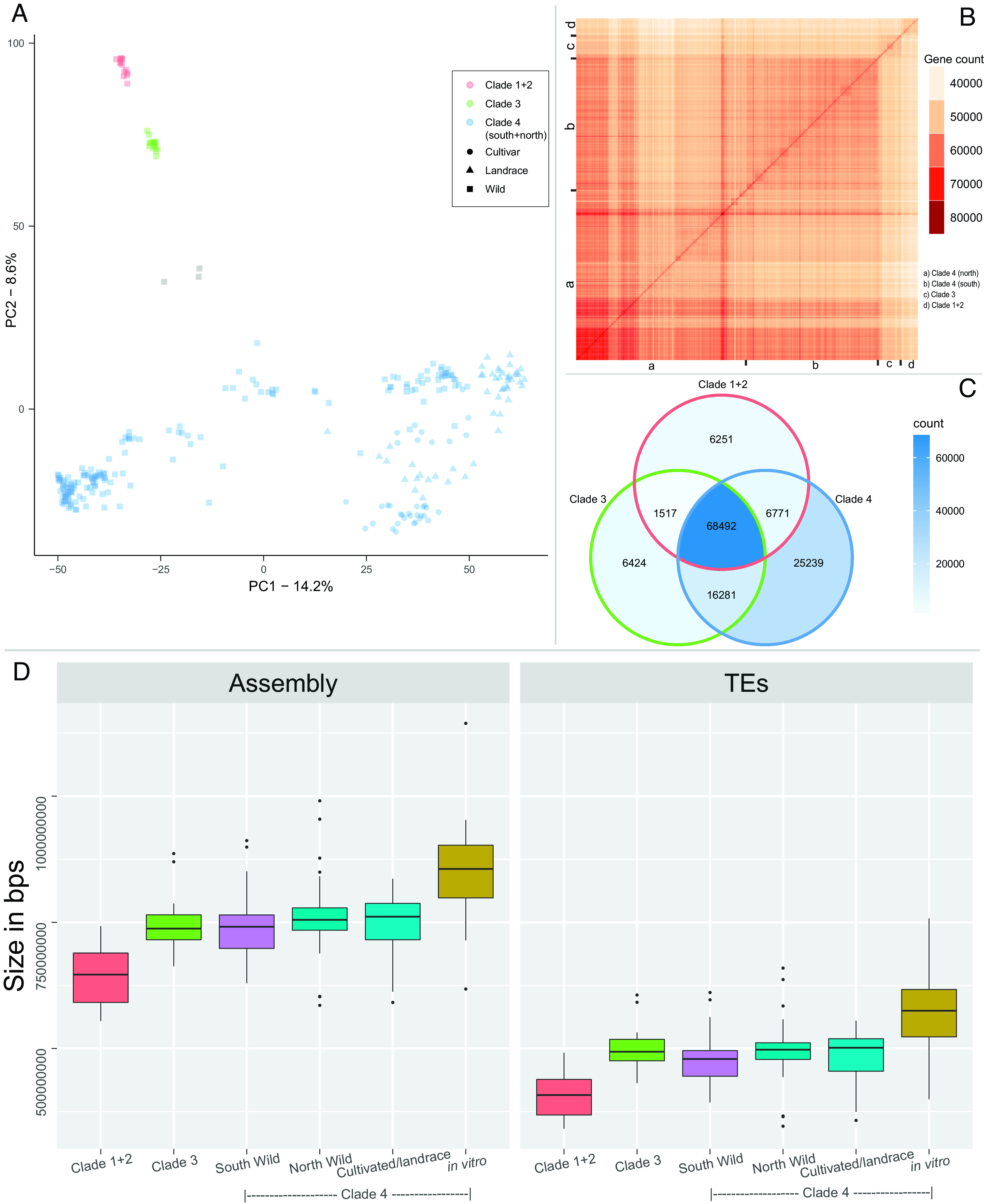
Analysis of different clades and subgroups in the pangenome of the *Solanum* section *Petota*. (*A*) A PCA based on the PAVs shows the clustering of accessions. (*B*) Heatmap showing the shared gene count among all the accessions. The accessions are ordered based on the phylogenetic tree. (*C*) Venn diagram of genes present in three clades: clade 1+2, clade 3, and clade 4. A gene is considered as present in a clade if at least one accession from that clade has it. (*D*) Boxplot of de novo genome assemblies and the amount of TEs between the specified groups.

Principal component analysis (PCA) of the PAVs also showed a separation similar to the one in the phylogenetic tree (Fig. 4*A*). A clear separation of clade 1+2, clade 3, and clade 4 species was observed, with the *S. sogarandinum* and *S. cajamarquense* accessions as a separate group. The southern wild species formed a separate cluster away from the rest of the clade 4 species. A close clustering including the wild clade 4 north species and the cultivated species is observed. The subsequent analyses exclude the *S. sogarandinum* and *S. cajamarquense* accessions from clade 3.

### Variation in Gene Content Distinguishes Clades and Subgroups.

To understand the differences in shared gene count among the accessions, a heatmap was generated showing the number of genes an accession has in common with the other accessions (Fig. 4*B*). For each of the three clades, the accessions within a clade have more genes in common compared to accessions between clades (*SI Appendix*, Fig. S10*A*), with mean shared gene counts in clade 1+2, clade 3, and clade 4 being 49,939, 52,105, and 54,953, respectively. The PAV data were also used to compare the gene content between the three clades, clade 1+2, clade 3, and clade 4. There are 68,492 genes that at least one member of these clades has, while 6,251, 6,424, and 25,239 genes are uniquely found in clade 1+2, clade 3, and clade 4, respectively (Fig. 4*C*). Similarly, comparing the clade-specific core gene content showed that there were similar functions shared between the core genomes of each clade and that there were 5,612, 3,880, and 2,892 unique core genes present in clade 1+2, clade 3, and clade 4, respectively (*SI Appendix*, Fig. S10*B*). The clade 1+2 unique core genome GO (GeneOntology) terms indicate enrichment in cold acclimation (GO:0009631) along with functions in regulation of abscisic acid (GO:0009787) and inositol metabolism (GO:0019310), which are related to the cold response (*SI Appendix*, Fig. S11). The clade 3 unique core genome is enriched for GO terms involved in Ultraviolet (UV) (GO:0009411), cold response (GO:0009409), and polyamine transport (GO:0015846) related to stress response, in addition to triterpenoid biosynthesis (GO:0016104), which produces defense metabolites including glycoalkaloids. The clade 4 unique core genome is also enriched for genes involved in triterpenoid biosynthesis and genes involved in positive regulation of transcription. Additionally, clade 4 unique core genes are annotated as regulating flower development (GO:0009909), which are also annotations for genes functioning in regulation of tuberization.

The PAV gene content was also used to discover the differences between the subgroups: wild species in clade 4 south (South Wild), wild species in clade 4 north (North Wild), the group with accessions with a history of in vitro propagation (in vitro), and the group with the remaining cultivated accessions (Cultivated/landrace) with no history of in vitro propagation. *SI Appendix*, Fig. S12*A* shows that there are 3,947, 316, 3,037, and 2,547 genes that are unique to the South Wild, North Wild, Cultivated/landrace, and in vitro group, respectively (at least one individual of that group has it). There are 950 core genes that are shared among all the groups except the in vitro conserved accessions and 768 genes that are only present in the core genome of the in vitro subgroup (*SI Appendix*, Fig. S12*B*). Functional enrichment of the in vitro unique core genes revealed functions related to transposition, DNA integration, RNA modification, and DNA recombination (*SI Appendix*, Fig. S13*A*). The genes absent from the in vitro group but present in the remaining groups were enriched in functions related to riboflavin biosynthesis, DNA metabolic process, regulation of cell shape, and protein secretion (*SI Appendix*, Fig. S13*B*).

### TE Content in the Pangenome.

The pangenome is composed of 75.5% Tes, and the analysis of gene content indicated functional enrichment of genes for DNA methylation and TEs in the cloud genome and in vitro subgroup (*SI Appendix*, Figs. S5 and S13*A*). The member accession assemblies were scanned for TE content and presence of transposition-related genes (Fig. 4*D*). There was no bias from the assembly quality in the number of TEs identified (*SI Appendix*, Fig. S14). Clade 1+2 accessions have very low numbers of TEs and TE-related genes (Fig. 4*D* and *SI Appendix*, Fig. S15) compared to the accessions from other groups. In particular, the accessions in the in vitro subgroup have greater numbers of TEs. The majority of the TEs in the pangenome are retrotransposons, especially Gypsy/DIRS1. The amount of retroelements and DNA transposons in the in vitro subgroup is much higher than in the clade 1+2 group (*SI Appendix*, Fig. S16). On the other hand, there are more LINEs in the clade 1+2 group compared to the other groups, though LINEs represent only a small fraction of TEs in all the accessions. Small RNAs are involved in gene regulation including TE silencing, and they are also more abundant in clade 1+2 compared to the other groups. Additionally, the *S. okadae* (OKA15), *S. commersonii* (COM), and *S. chacoense* (M6) accessions, which were in vitro propagated, have a higher TE gene count compared to the rest of that clade 4 south group.

## Discussion

Crop pangenomes have impacted cultivar improvement through advancing understanding of the importance of structural variation of the genome (36). A recent tetraploid potato pangenome provided insights into domestication in potatoes and the influence of wild species via extensive introgression to cultivated potatoes (21) and a recent diploid potato pangenome that incorporated 44 species plateaued in terms of gene content at about 40 genomes (23). The current study extends the pangenome to the larger *Solanum* section *Petota*, which includes tuber-bearing landraces and wild species of all ploidy levels. This larger panploidy pangenome plateaus at about 80 genomes and showcases a vast genetic diversity in the section, which includes species with asexual and sexual reproduction, self-incompatibility, introgression, and interspecific hybridization complicated by pre- and postzygotic reproductive barriers (24). The *Solanum* section *Petota* pangenome that was constructed includes publicly available and novel genomes selected from our previous studies that cover a wide geographical range and taxonomic diversity (2, 3). This pangenome is complete based on BUSCO assessment (99% and 100% for *Solanales* and *Viridiplantae,* respectively) compared to single-potato reference genomes that range from 83.9 to 97.9%. Utilizing the pangenome to make genome-wide comparative analysis, structural variation detection, and functional analyses greatly facilitates understanding of speciation in *Petota* and provides genomic resources to enhance improvement of the potato crop through introgression with beneficial wild species. Analysis of PAV of cultivated vs. wild accessions demonstrated that domestication was associated with changes in photosynthesis, defense response, and metabolic processes. Biological functions related to root development and dormancy were also noted differences.

We report a PAV-based phylogenetic tree and observed groupings based on shared gene loss/gains. The pangenome phylogenetic tree constructed utilizes genome-wide PAV of genes, including TEs, to determine genetic distance for *Solanum* section *Petota* and compared the largest collection of nuclear genomes from this section to date. Gene duplication with PAV involves DNA repair processes as well as TE activation, which not only affect their own copy numbers when mobilized but also other genes (37). PAVs are an important source of genetic variation, evolutionary history, and interspecies differences. The present study shows an increase in gene content in landraces and cultivars compared to wild species suggesting a trend of gene gain during domestication in potato, as opposed to other plant species (27, 33). Moreover, an increase in the gene content among the polyploids shows the diversity that exists within section *Petota*.

Processes leading to PAV can also affect SNPs and indels. Hence, there were common features of this PAV phylogenetic structure to others that were derived from SNPs and indels (14, 15, 38), and it supports the cladistic groupings of Spooner (2). Incorporating different ploidy levels in the PAV-based analysis showed that the diploid landraces (*S. phureja*, *S. stenotomum*, and *S. goniocalyx*) have similar gene PAVs as the lumped *S. candolleanum* species (3), whereas the *S. tuberosum* cultivars (including *“Andigena”* and *“Chilotanum”* groups) species have a diverse genetic base and share similarities with the southern and northern wild species. The tetraploid *S. tuberosum* cultivars have shown introgressions from a large number of wild species (14, 21). Another added advantage of the PAV information was the capacity for functional analysis of groupings found in the phylogeny, which uncovered findings on speciation in the complex *Solanum* section *Petota.*

The clades recovered here and in previous studies have a strong geographic identity, with clade 1+2 species found predominantly in North and Central America, clade 3 in Ecuador and northern Peru, and clade 4 predominantly in South America (2). Genes unique to each clade were found to have functions associated with abiotic stress responses, particularly cold, suggesting PAV involvement in adaptation to the environment and a contribution to separation of species in the clades. Additionally, genes involved in triterpenoid biosynthesis, which produce defense compounds including precursors for steroidal glycoalkaloids, were also found to be unique to clades 3 and 4. These results concur with the variation in steroidal glycoalkaloids observed in *Solanum* section *Petota* in other studies (39). Clade 4 also had unique genes annotated for functions in flower development. Regulators of tuberization in potatoes are homologs of flowering regulators and also carry flower development annotations (40, 41). The results indicate that PAV of genes involved in flowering/tuberization contribute to the grouping in clade 4. Our results are supported by other studies showing selection of genes involved in glycoalkaloid production and tuberization in domestication (14, 15, 21).

The core genome of the *Petota* pangenome across clades demonstrates fundamental biological, molecular, and cellular functions. The shell genome notably has enrichment for genes in secondary metabolite biosynthesis involved in the production of steroidal glycoalkaloids that are characteristic defense metabolites of the genus *Solanum.* The cloud genes show functions in response to stress and TE activity. Hoopes et al. (21), also noted TEs for the cloud and high copy number genes of the tetraploid potato pangenome (21). The high TE content in the *Petota* pangenome indicates that TEs are an important factor to consider in genome evolution for the section and likely the entire genus. TEs are known to affect genome structure and function leading to gene disruptions, genomic rearrangements such as translocations, deletions, duplications of genetic elements along with changes in gene expression such as gene inactivation, gene dysfunction, and differential expression (42, 43). These elements are typically suppressed by intricate regulatory machinery in genome defense mechanisms (43, 44). For instance, DNA methylation can suppress TE activity to avoid potential negative effects on a plant’s normal development (45). Mutations induced by TEs can be eliminated in a population or spread throughout depending on the effective population size (43). Estimating the rate of transpositions in a genome is a challenge; however, it is thought to be orders of magnitude higher than nucleotide base mutations making TEs a major player in evolutionary change (42). Plants face numerous biotic and abiotic stresses and TEs can play a role in launching genetic plasticity by altering gene expression (43) and driving bursts of TE activity (44).

TEs affecting potatoes have been previously demonstrated in the literature. A TE excision from a gene encoding flavonoid 3′, 5′-hydroxylase was documented as leading to a tuber skin color change. This was caused by a stowaway Miniature Inverted-repeat Transposable Element (MITE) which was excised from the potato genome under tissue culture conditions producing somaclonal variants leading to a reversion of flavonoid 3′, 5′-hydroxylase function and a purple-skinned tuber (46). The potato genes controlling self-incompatibility, *Sli* (47, 48), and photoperiod-dependent tuberization, *StCDF1* (41), are functionally disrupted by TEs leading to new traits, the former providing capacity for selfing and the latter enabling natural expansion of section *Petota* further south in South America to regions with long day lengths and adoption of potato for cultivation in northern temperate zones. The current study provides additional evidence for a role for TEs in speciation in section *Petota*. TE mobilization is connected to limitation in gene flow between nascent species (49). In section *Petota,* which is one of the most rapidly diversifying or radiating sections within *Solanum* (50) and where many younger species resulted from direct hybridization of two species (2), TE mobilization may have contributed to the diversity and the large number of younger species observed.

Clade 1+2 species had a lower number of TEs and differences in classes of TEs compared to other clades (*SI Appendix*, Fig. S17). Based on taxonomy, Hawkes (3) proposed that the wild species in *Petota* originated in North and Central America. It was further proposed that expansion proceeded southward toward South America with species spreading both further southward and back northward. The *Petota* pangenome suggests that TE activation may have contributed to the genome and taxonomic diversity observed in *Solanum* section *Petota* species that adapted to new environments while expanding southward from North and Central America, though other explanations such as genetic drift are also possible for the diversity.

Clade 1+2 species also have hybridization incompatibility with other clades of *Petota* (51–53). Hybridization barriers in *Solanum* can be explained through the endosperm balance number (EBN) concept (54) with assignment of 1EBN to clade 1+2 species. These are incompatible with clade 3 and 4 species, which are 2EBN and 4EBN. DNA methylation plays an important role in species hybridization barriers in *Arabidopsis thaliana* (55–61) and wild *Solanum* relatives of tomato (59), where it controls the uniparental gene silencing in the endosperm in genomic imprinting. Loss of DNA methylation and genomic imprinting deregulates uniparental gene silencing causing inappropriate gene dosages leading to underdevelopment of endosperm and seed abortion. The results of the current study show low levels of TE for clade 1+2 species, which all have 1EBN, compared to clades 3 and 4, which both have 2EBN and 4EBN species. Given that DNA methylation is involved in TE silencing (49), it is intriguing to consider a possible role for TEs, DNA methylation, and genomic imprinting in section *Petota* EBN reproductive barriers; however, further studies will be needed to clarify the connection.

Due to its high heterozygosity levels and outcrossing ability along with the need to ensure consistency and uniformity of quality traits, potato is typically vegetatively propagated (62). Many *Solanum* section *Petota* species have also been readily adapted to in vitro propagation through conservation at genebanks (63), as this is one of the most effective methods of conserving potato to ensure that allelic states are fixed and to keep them virus-free; yet, they are also stored as seed to help preserve the diversity contained within the species. Conservation as true seed, in addition to tissue culture, is a consideration for breeding materials and genebanks, given the variation of TE observed in the current study. Based on a source material history that includes in vitro tissue culture conservation and propagation, accessions were subgrouped into an in vitro accessions subgroup, which consisted of cultivated *S. tuberosum* varieties and breeding germplasm along with landraces and in vitro accessions from wild potato species. The highest numbers of TE-related genes were found in this in vitro subgroup compared to the rest of the accessions in the pangenome. Because the common denominator in this group is source material from in vitro culture, rather than relatedness, the results suggest that in vitro culture triggers TE activation, which was also found by others (64). The current study provides evidence that these changes occur in *Solanum* section *Petota* and provides further evidence for TE activation in adaptation to new environments. Transposition, is thus, a source of genetic variation that can be used for breeding. The *Petota* superpangenome provides a foundation for building tools to further investigate and apply TEs to climate-smart potato breeding.

## Conclusions

The future survival of the potato crop in the face of climate change is dependent on our conservation of biodiversity and our understanding of how to introgress diversity into current and developing cultivars. Here, we present a comprehensive pangenome constructed from 296 accessions representing 60 species in the *Solanum* section *Petota*. Analysis of the pangenome provided evidence for a role of PAV, particularly TE variation, in speciation in *Solanum* section *Petota* and uncovered a trend for increased TE-related content in in vitro propagated material, akin to the increase in TE activity in natural adaptation due to stress.

## Materials and Methods

### Genome Sequences of Potato Accessions.

A total of 296 potato accessions were used in the construction of the *Solanum* section *Petota* pangenome. These represent 60 species based on the original sources: 63 accessions are from a diverse panel that includes wild species, landraces, and cultivars (14); 199 accessions include mostly wild *Solanum* species and potato cultivars with a broad geographical origin (15); 13 accessions from the International Potato Centre (CIP), Lima, Peru (17, 18, 65, 66); ten accessions from Agriculture and Agri-Food Canada (AAFC) (67, 68); six tetraploid potato cultivars from Europe and North America (21); and five published reference genome sequences (13, 16, 19, 20, 69). A detailed list of these accessions is presented in *SI Appendix*, Table S2. The taxonomy is according to the respective original source.

Because an accession is typically defined as a group of related plant material, some genetic variability can exist within a seed accession consisting of seeds from a cross that created a new combination of alleles. In potatoes, seed accessions stored in genebanks are typically heterogeneous material that was collected from its native environments or regenerated in a controlled manner. In clonally propagated material, an accession is a single individual or genotype in which the allele combinations are fixed through vegetative propagation. The genomes in this study included wild seed material in which a single seed from an accession was chosen for analysis, in vitro propagated accessions in which a single plant (clone) represented the accession, and tuber propagated accessions, which includes varieties and breeding lines with a previous history of in vitro propagation for clonal maintenance and/or certified seed production. In all cases, there is a single genotype representing the accession.

### Construction of the Pangenome.

The haplotype-resolved reference genome of diploid potato, *S. tuberosum* group Tuberosum RH89-039-16 v3 assembly (19), was used as a reference for the construction of the *Petota* pangenome. In various previous studies, de novo genome assemblies were constructed for 22 accessions, which were used directly in constructing the pangenome, with remaining accessions being assembled de novo using raw sequencing reads. The 11 novel accessions *S. bukasovii* (BUK2), *S. okadae* (OKA15), and the nine diploid clones from the AAFC germplasm collection (H412-1, W5281.2, 10908-06, DW.84-1457, 12625-02, 12120-03, 08675-21, 07506-01, and 11379-03) were sequenced and assembled as described (66, 68). The raw sequencing reads of the remaining 262 accessions were downloaded from the National Center for Biotechnology Information's (NCBI) SRA submission portal, trimmed using *Trimmomatic v0.39* (70) and de novo assembled using *Megahit v1.2.9* (71). Different *k* values were used for datasets with different read lengths, *‘--k-min 21 --k-max 85 --k-step 8’* was set for 100-bp read lengths, ‘--k-min 25 --*k-max 95 --k-step 10’* was set for 125-bp read lengths, *‘--k-min 31 --k-max 111 --k-step 10’* was set for 150-bp read lengths, and *‘--k-min 35 --k-max 125 --k-step 10’* was set for 350-bp read lengths. The resulting contigs with <500-bp length were filtered out from the assemblies.

To remove sequences for organellar genomes from the assemblies and keep only nuclear sequences, the RH genome assembly was concatenated with publicly available potato organellar genomes (67, 72–74). Each of the downloaded and newly assembled sequences was aligned against the RHO (RH plus organelles) with *nucmer* aligner from the *mummer v4.0.0beta2* and with the ‘*--maxmatch*’ option to get maximal matches (75) (*SI Appendix*, Fig. S18). The matches were filtered with a minimum of 300-bp alignment length and 90% identity using *delta-filter*. Sequences that did not align with the RHO were kept as unaligned sequences. Any aligned sequences containing a continuous unaligned region of at least 500 bp were added to the unaligned sequences.

The unaligned contigs were filtered for contaminants by querying against the NCBI nonredundant nucleotide database (downloaded on 07-07-21) with a minimum identity of 90% using *blast+ v2.11.0* (76). The contigs with only hits to green plants were called reliable and kept, whereas those contigs with only hits to other than green plants were called contaminated and removed. The contigs with hits to both the green plants and others were kept when at least 90% of the query-covered region matched green plants. Furthermore, the unaligned contigs were also checked for adaptor and remaining organellar sequences by querying against the *univec* database and organellar sequences from complete *Solanum* taxa. After the blast filtering, *nucmer* was used iteratively to compare individual accessions and removed further redundancy in the unaligned contigs to get a single unaligned sequence file. The clean unaligned sequences were further checked for redundancy by running an all vs. all alignment using *blast+* followed by *cd-hit v4.8.1* (77). A sequence identity threshold of 95% and query coverage of 95% were used for the above *nucmer*, *blast+*, and *cd-hit* steps. Any organellar sequences present in the RH assembly were removed, and only the nuclear sequences were concatenated with the unaligned nonredundant sequences to generate the *Petota* pangenome. The quality and completeness of the pangenome were assessed using *QUAST v5.0.2* (78) and *BUSCO v5.2.2* (79).

### Annotation of the Pangenome.

A custom repeat library (CRL) was constructed for the pangenome using *repeatmodeler v2.0.2a* (80), and the unknown repeats were annotated using the transposase database. Also, gene fragments from the unknown repeats were removed using *ProtExcluder* (81) to create a final CRL. The repeats in the unaligned sequences of the pangenome were soft-masked using *RepeatMasker v4.1.1* (82). The final CRL of the pangenome and DMv6.1 (69) were used as the input repeat libraries for RepeatMasker. The soft-masked unaligned sequences were annotated with the BRAKER pipeline (83, 84), using the reference protein sequences and the ribonucleic acid sequence (RNA-seq) data. For the first run, protein sequences of *Solanales* from the OrthoDB were concatenated with the DMv6.1, RH, and potato pantranscriptome (31) protein sequences. An ab initio gene prediction was performed by *AUGUSTUS* (85) and *GeneMark-EP* (86) with the protein sequences as extrinsic evidence. For the second run, RNA-seq data from 102 libraries were downloaded from the NCBI (*SI Appendix*, Table S3), trimmed using *TrimGalore v0.6.7* (87) and aligned against the unaligned pangenome using *hisat2 v2.2.1* (88). The resulting binary alignment map (BAM) files were merged using *Samtools v1.13* (89). The gene prediction was performed by *AUGUSTUS* and *GeneMark-ET* (90) with the merged bam file as RNA-seq evidence.

The results from both the runs of BRAKER were combined using *TSEBRA v1.0.3* (91). The resulting annotations were filtered using *gFACs v1.1.2* (92) with the following parameters *‘--rem-all-incompletes --rem-genes-without-start-codon --rem-genes-without-stop-codon --allow-alternate-starts --min-exon-size 20 --min-intron-size 20 --min-CDS-size 74 --statistics --create-gff3 --allowed-inframe-stop-codons 0*’. Gene models with >50% repeats in its coding region were removed using *gffcompare v0.12.6* (93). Format errors in the gff3 file were corrected using *GFF3toolkit v2.1.0* (94) to create the final set of unaligned pangenome annotations. The genes were also functionally annotated by *ahrd v3.3.3* (available at https://github.com/groupschoof/AHRD) to get human-readable descriptions of the annotations by querying against the TrEmbl, UniProt, and Araprot11 database (downloaded on 2021-12-23). *Pfam* domains and GO terms were identified in these genes by searching against the InterPro’s database using *interproscan v5.52-86.0* (95). Finally, the annotation of the unaligned pangenome was merged with the RH genome annotation as a complete annotation of the pangenome.

### PAV Analysis.

The filtered short-read data from 285 accessions were used for the PAV analysis. Eleven accessions were excluded from this analysis due to either short read length (≤100bps) or hybrid origin. The reads were mapped individually to the pangenome using *bwa v0.7.17* (96). Only the mapped and properly paired alignments (forward and reverse reads mapped at expected distance and orientation) were kept for further analysis using *Samtools* with an -f2 flag. *SGSGeneLoss* was used to call PAV from the alignments (97). Genes were considered absent if less than 20% of its exon regions were covered by a minimum of n reads, where n was defined based on average depth against the pangenome (n = 2, 4, 6, 8, or 10 when depth = 0 to 10, 11 to 20, 21 to 40, 41 to 80, or 80+, respectively). These settings were chosen to reduce potential bias in calling PAV, after testing different n values for varying sequencing depths. All the genes from the pangenome were classified into three categories based on their PAV results. If a gene is present in 97% of the accessions, it is called a core gene, similarly, shell and cloud genes were categorized if they are present in 3 to 97%, and <3% of the accessions, respectively. The PAV data were converted into binary data and used in the construction of a phylogenetic tree using *IQ-TREE2 v2.1.3* (98). A best-fit substitution model was determined by *IQ-TREE2*, and a maximum-likelihood phylogenetic tree was inferred with a substitution model GTR2+FO+R10 and 1000 bootstrap replicates. PCA of the PAV was conducted using *R v4.1.0 prcomp* command with default parameters. GO enrichment analysis was performed to determine the enriched GO terms and their biological process using *topGO v3.14* (99).

### Analyzing Gene Frequency Differences.

To identify genes under selection, the following comparisons were made: clade 4 wild vs. landraces and clade 4 wild vs. cultivars to analyze differences in gene presence frequencies. A 2×2 contingency table was constructed for each gene in each group based on their PAV status. Fisher’s exact test was used to calculate the significance of the differences in gene presence frequencies between the compared groups. The *P*-values obtained from Fisher’s test were corrected using a false discovery rate (FDR). An FDR-corrected *P*-value <0.001 with fold change in gene occurrence frequencies >2 was used as the criteria for significantly enriched genes between the compared groups (27, 33). The genes with higher frequency in the landrace or cultivar than the wild species were considered “favorable genes,” and those with lower frequencies than wild species were considered “unfavorable genes”. Furthermore, the favorable genes with higher frequencies in landraces than cultivars were called landrace-favorable genes, and higher frequencies in cultivars than landraces were called cultivar-favorable genes.

## Supplementary Material

Appendix 01 (PDF)

## Data Availability

The raw genomic reads and de novo assemblies used in this study are publicly available, and each of the accession numbers is mentioned in respective tables (*SI Appendix*, Tables S2 and S3). All study data are included in the article and/or *SI Appendix*. The whole pangenome assembly is available through DRYAD (https://datadryad.org/stash/dataset/doi:10.5061/dryad.cfxpnvxbn) (100).
