# Supplementary material for: Pangenome analyses reveal impact of transposable elements and ploidy on the evolution of potato species
Source: Proc Natl Acad Sci U S A. 2023 Jul 24;120(31):e2211117120. doi: 10.1073/pnas.2211117120 (PMC10401005; doi:10.1073/pnas.2211117120)
Supplement: Supplementary file 1 — Appendix 01 (PDF) [file pnas.2211117120.sapp.pdf]

Supplementary Information for

Pangenome analyses reveal impact of transposable elements and ploidy on the evolution of potato species

Ilayda Bozan[a,1], Sai Reddy Achakkagari[a,1], Noelle L. Anglin[b,c], David Ellis[b], Helen H. Tai[d], Martina V. Strömvik[a,2]

[a]Department of Plant Science, McGill University, Sainte-Anne-de-Bellevue, QC H9X 3V9Montreal, Canada

[b]International Potato Center, Lima 15023, Peru

[c]United States Department of Agriculture - Agricultural Research Service USDA ARS Small Grains and Potato Germplasm Research, Aberdeen, ID 1691S 2700W

[d]Fredericton Research and Development Centre, Agriculture and Agri-Food Canada, Fredericton, NB E3B 4Z7 Canada

[2]To whom correspondence may be addressed. Email: martina.stromvik@mcgill.ca.

1 I.B and S.R.A contributed equally to this work

This PDF file includes:

Figures S1 to S18

Tables S1 to S3

Supplementary Figures

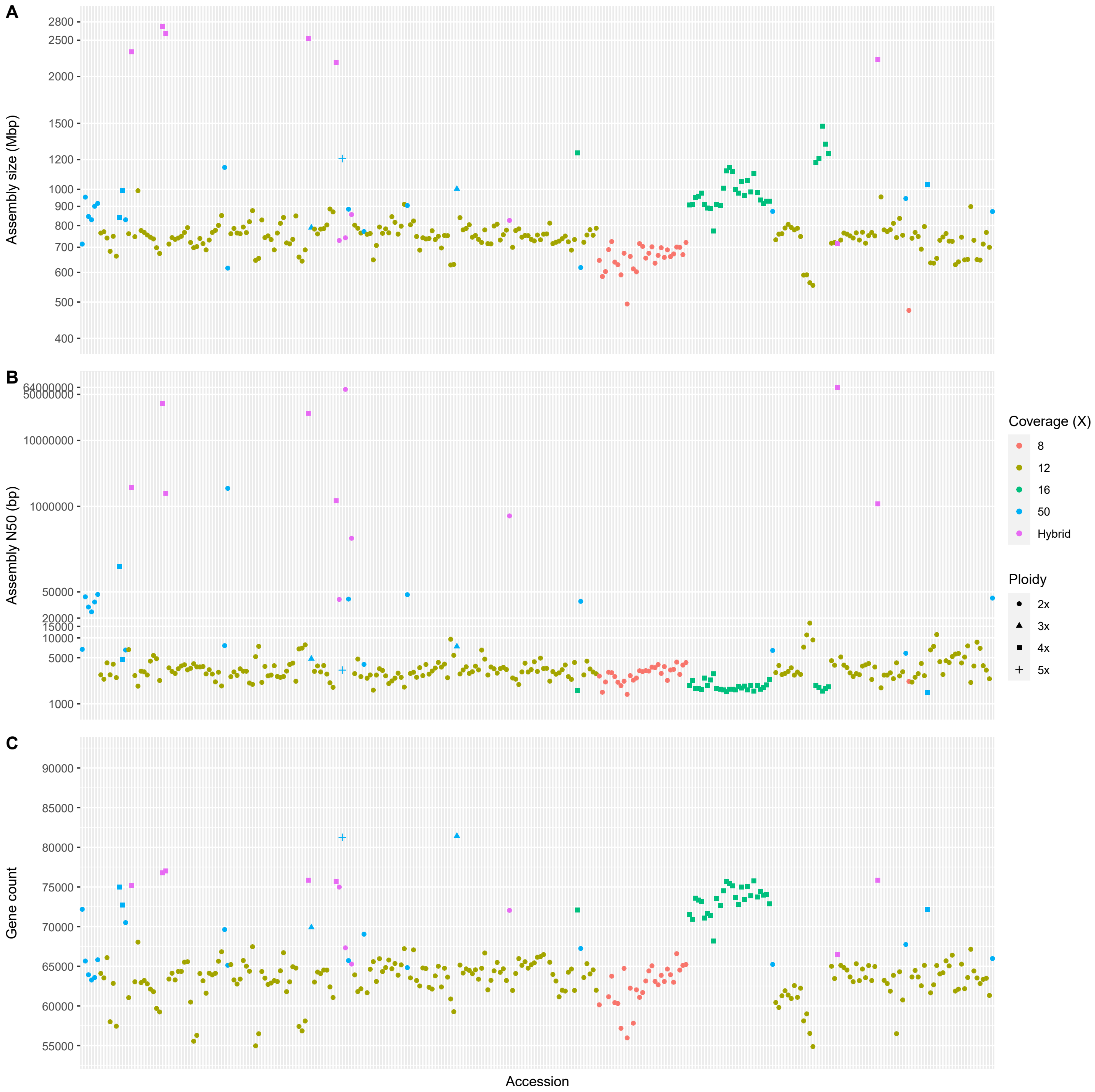

Fig. S1: Genome assembly statistics for each accession and its (a) assembly size (b) assembly N50 value and (c) their gene count

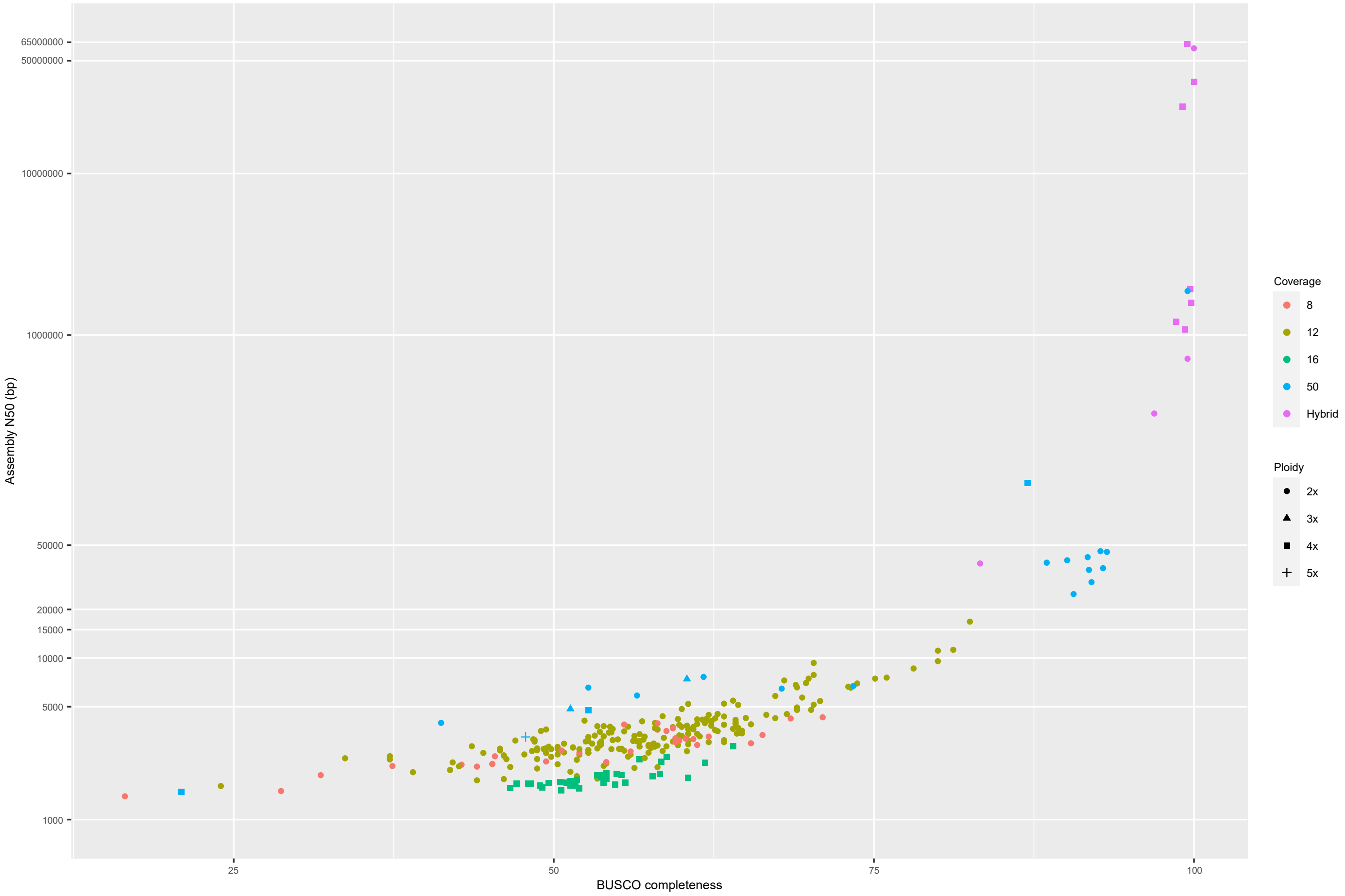

Fig. S2: Scatter plot of assembly N50 and BUSCO completeness

The two assembly statistics, N50 and BUSCO completeness, are plotted for each accession. The accessions with long-read assemblies and higher coverage have better N50 values and BUSCO scores compared to the short-read assemblies. Overall, a positive correlation between the N50 and BUSCO scores are observed.

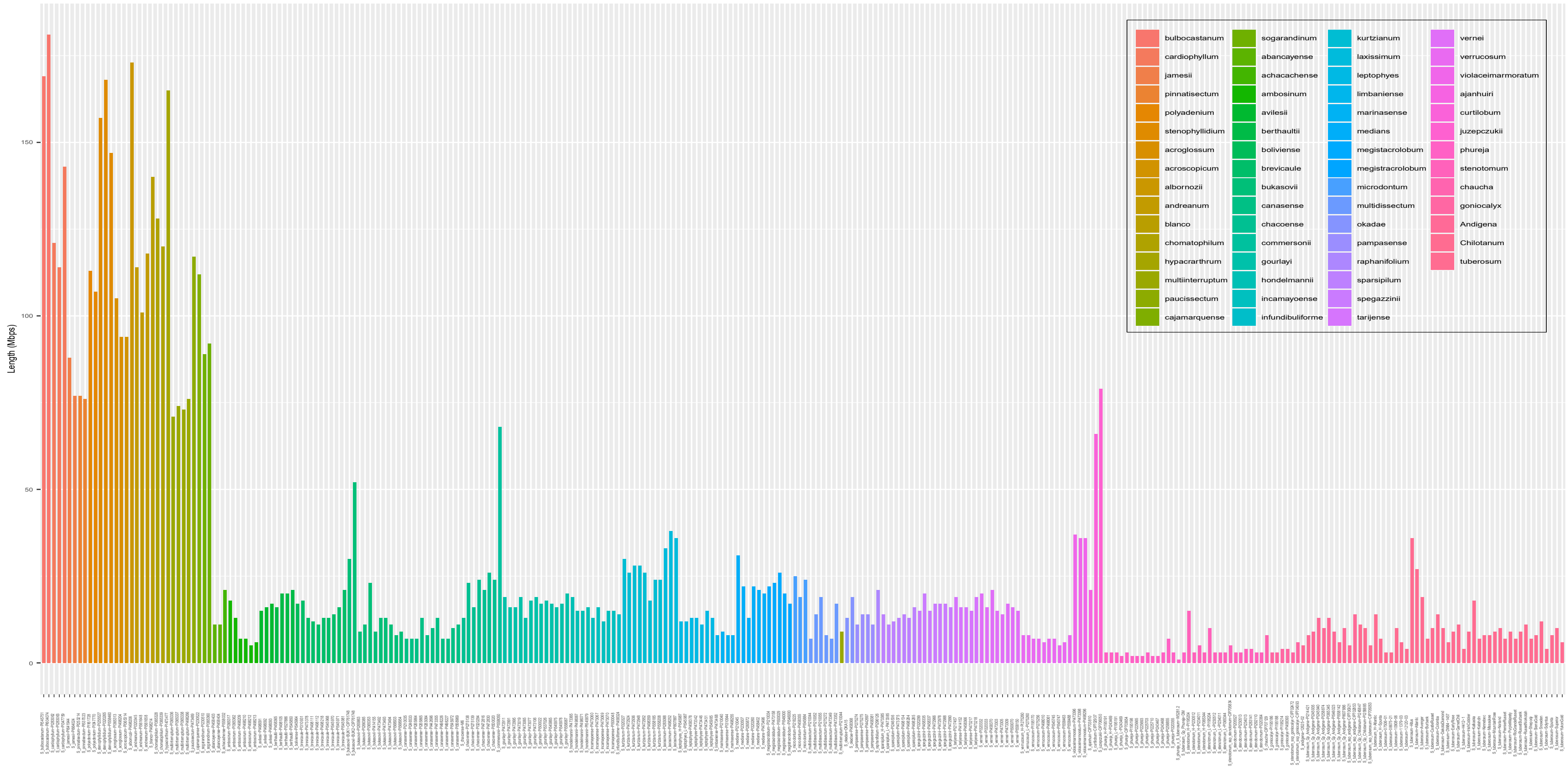

Fig. S3: Contribution of each accession to the non-reference sequences of the pan-genome

The total number of bases from each accession added to the pan-genome are shown here. The wild species, especially the Clade 1+2, and Clade 3 wild species, have contributed more sequences to the pan-genome than the cultivars or landraces.

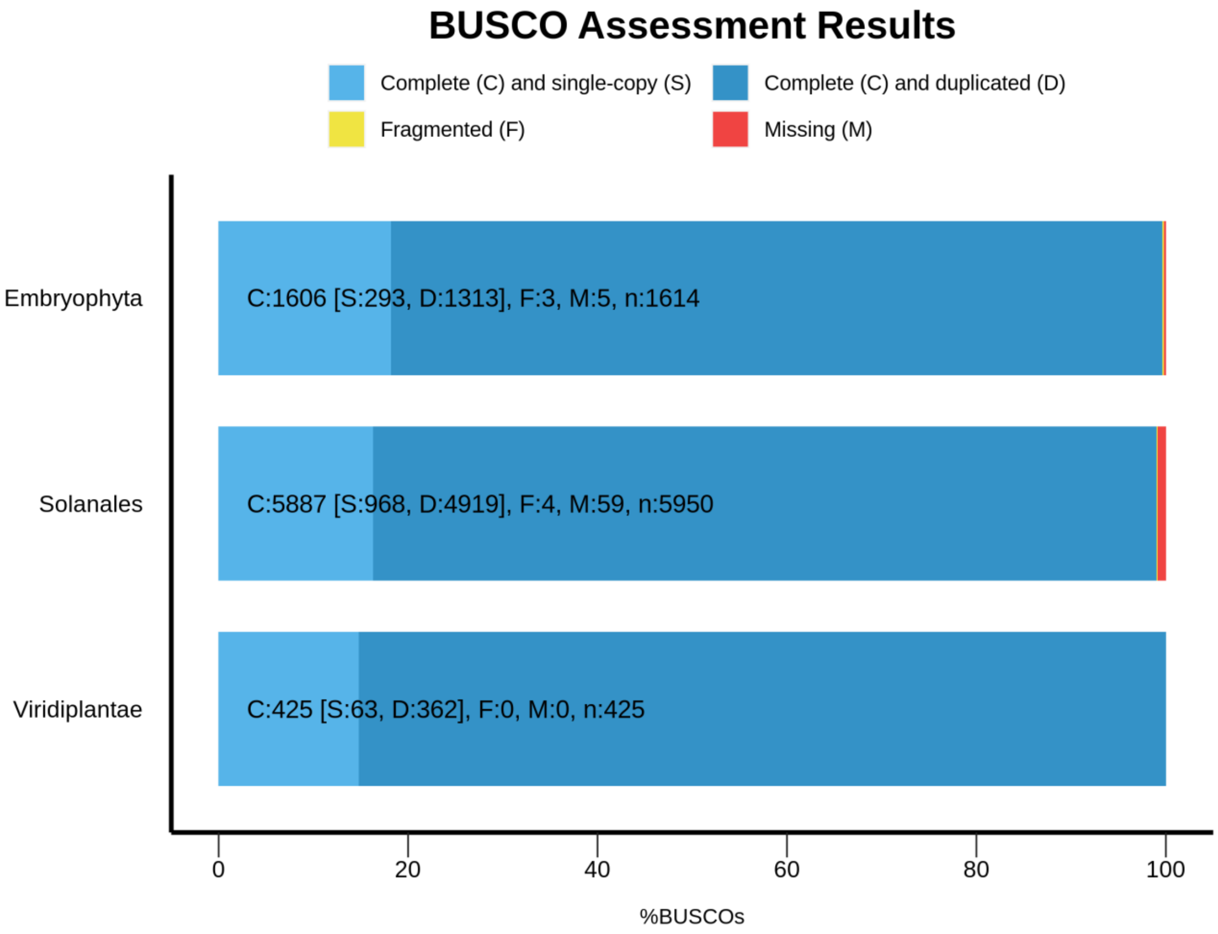

Fig. S4: The BUSCO assessment results of the *Solanum* section *Petota* pan-genome against the *Embryophyta*, *Solanales*, and *Viridiplantae* datasets.

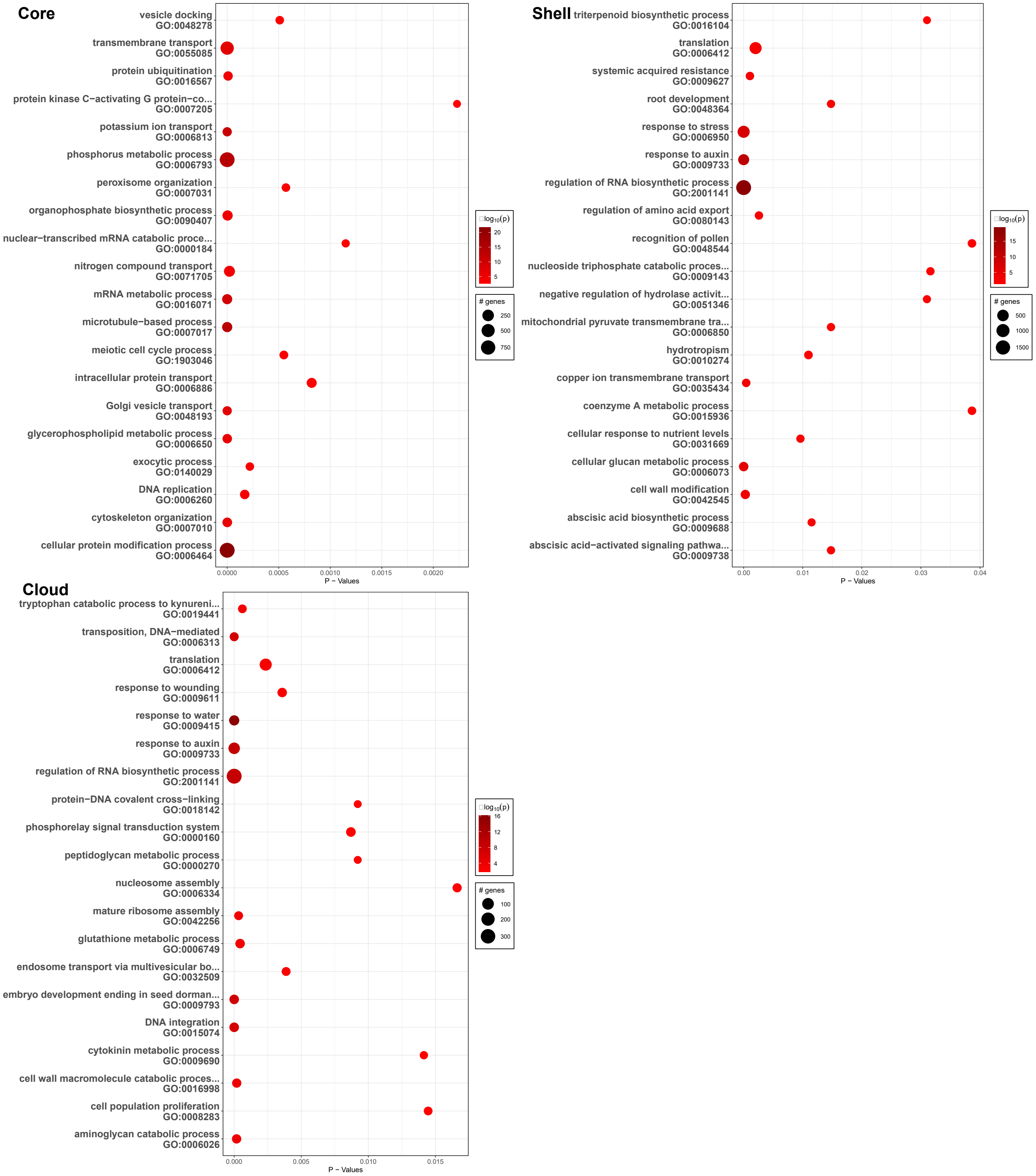

Fig. S5: Functional enrichment analysis of a) Core, b) Shell, and c) Cloud genome of the pan-genome

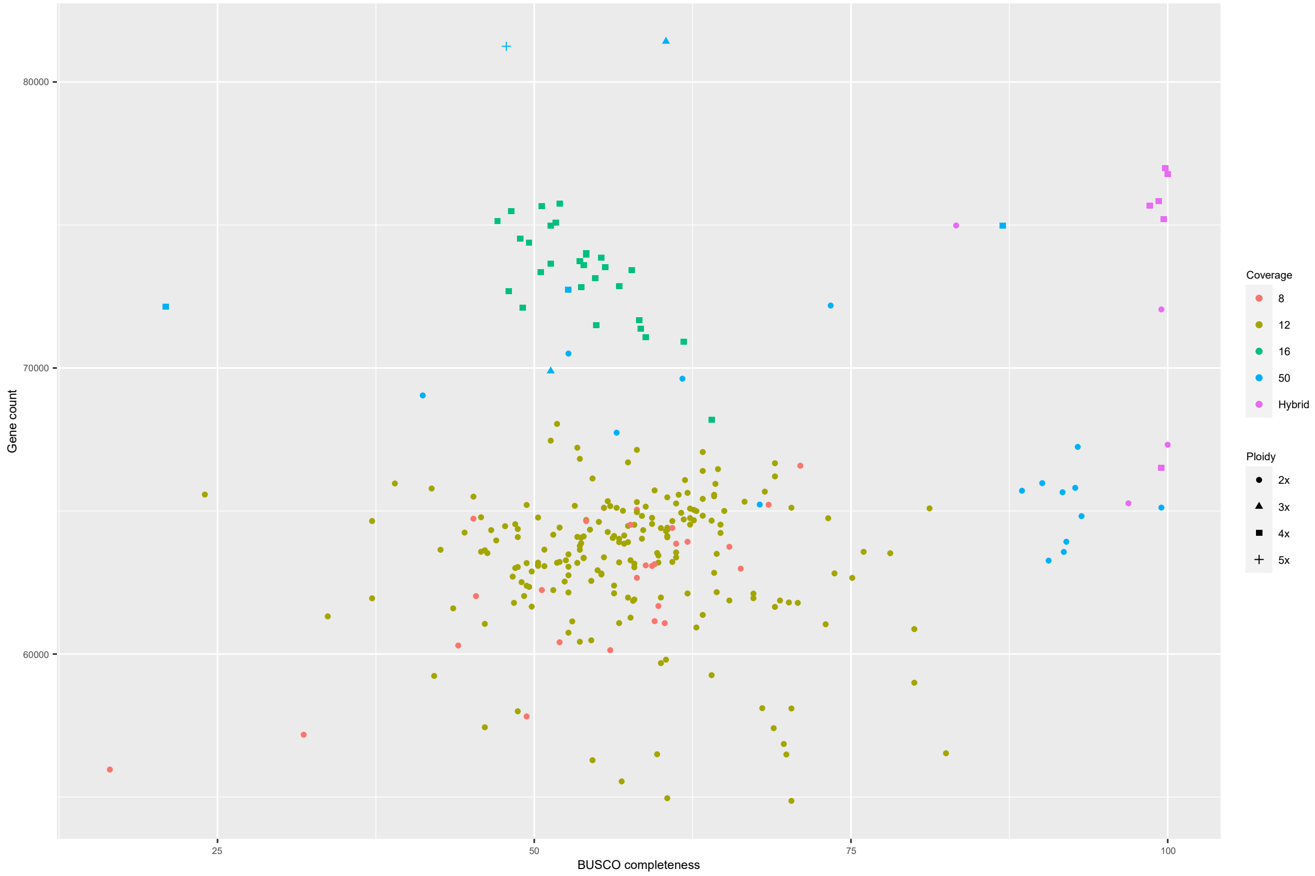

Fig. S6: Scatter plot of gene count against the BUSCO score.

There is no relation found between the completeness of the genome and their gene count. The accessions with highest number of genes have low BUSCO scores and the accessions with highest BUSCO scores have average gene count. The increase in gene count is mainly driven by ploidy.

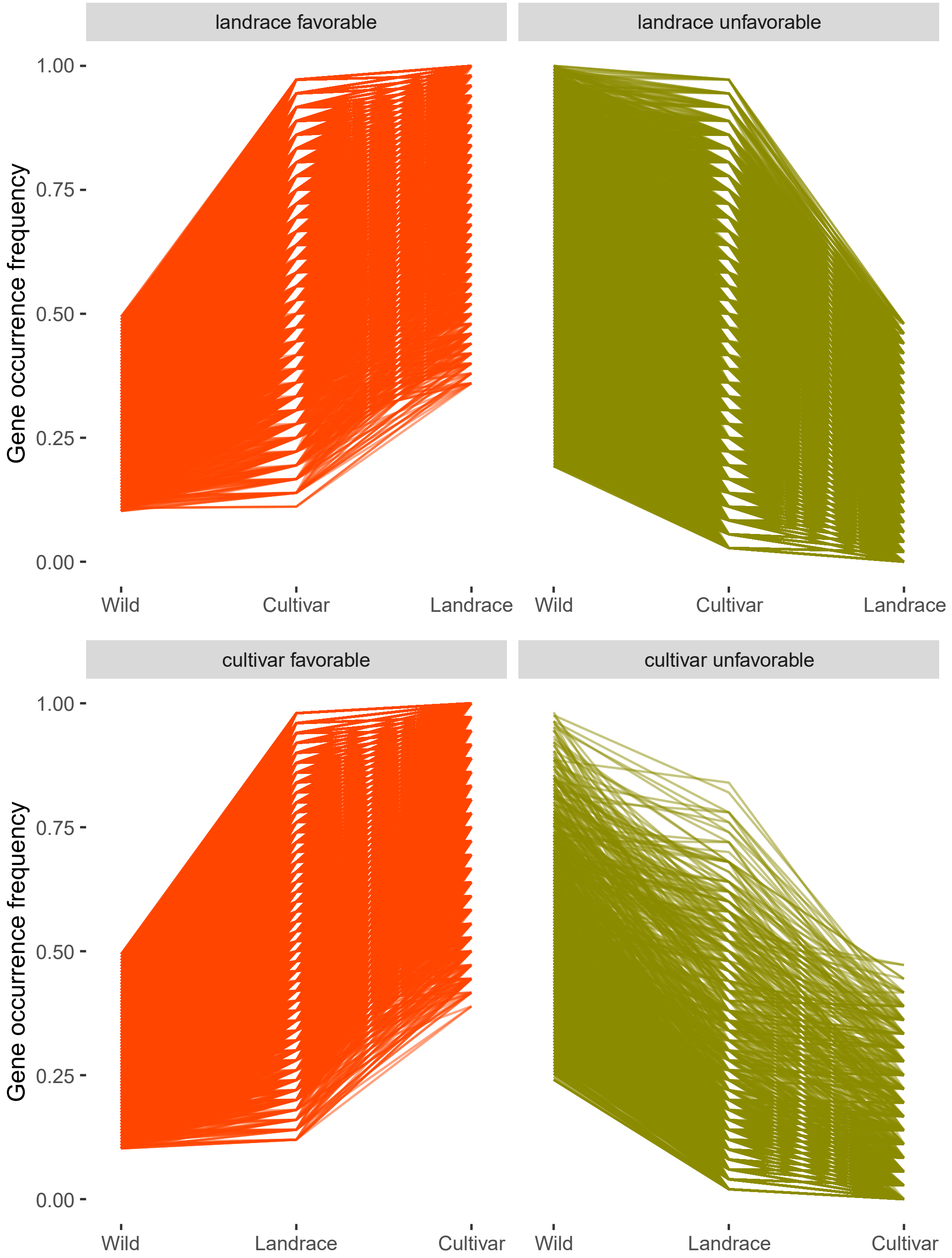

Fig. S7: The genes with significant differences in their occurrence frequency between the wilds and landrace/cultivars. The significant genes with frequencies higher than cultivars are called landrace favorable genes, whereas the higher frequency in cultivars than landraces are called cultivar favorable genes. Similarly, landrace and cultivar unfavorable genes are defined as genes with lower frequencies in landraces than cultivars and lower frequencies in cultivars than landraces, respectively.

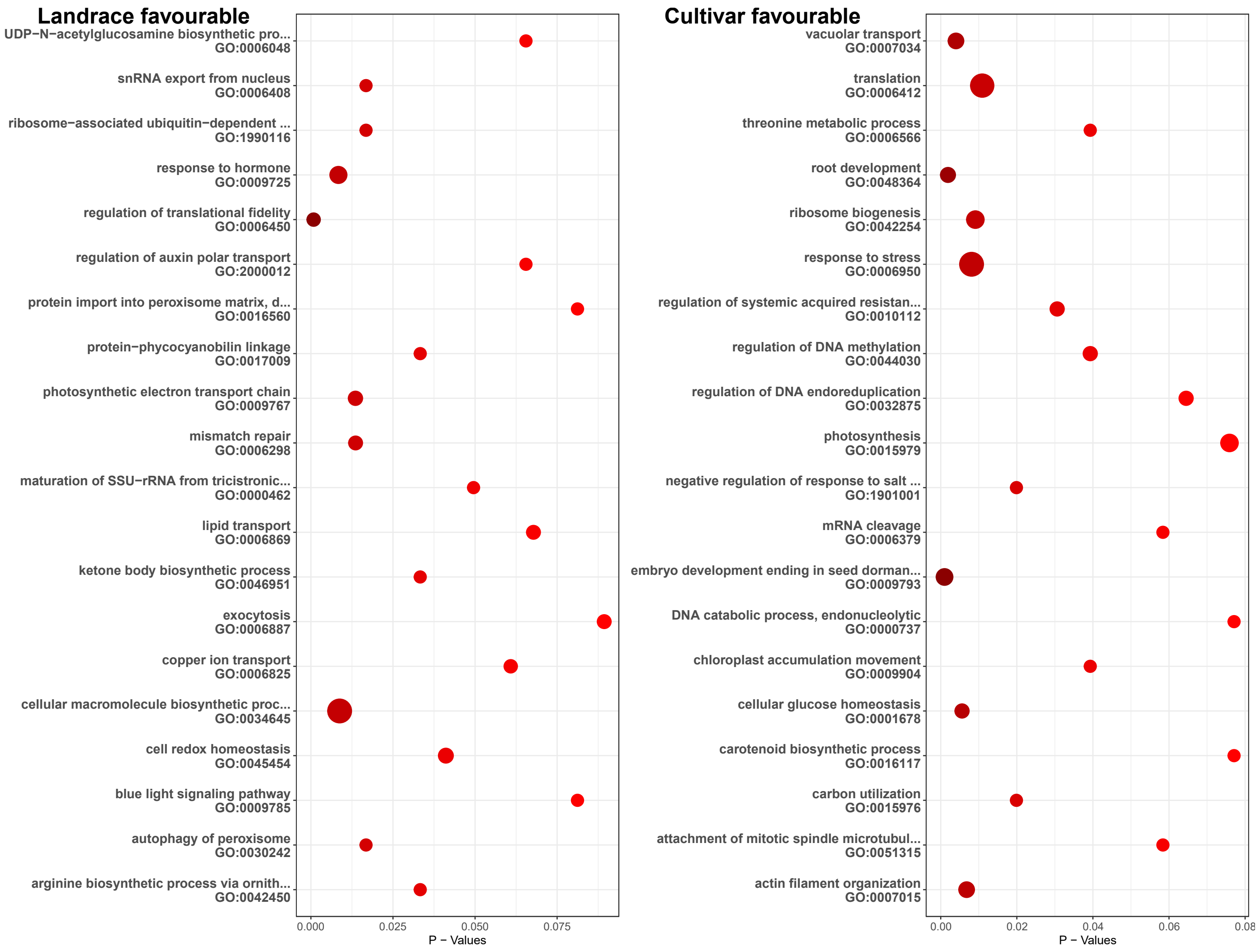

Fig. S8: The enrichment analysis of the landrace favorable and cultivar favorable genes.

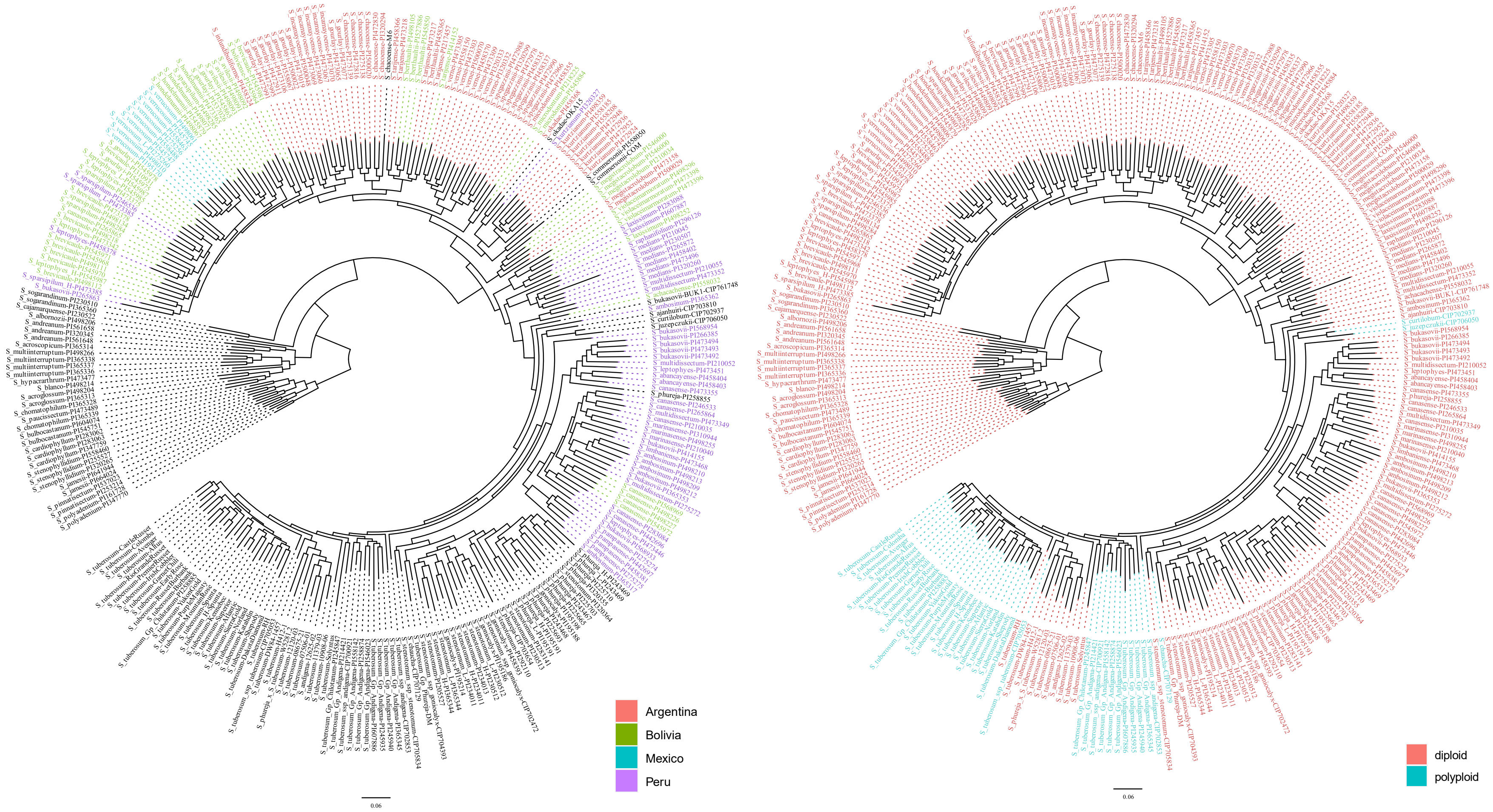

Fig. S9: The PAV phylogenetic tree with accessions colored based on their origin (left), and ploidy (right). The Clade 4 wild species are the only ones colored and we can see majority of the Clade 4 south accessions are from Argentina, Bolivia, and Mexico (*S. verrucosum*) and the Clade 4 north accessions are from Peru, corroborates with the previous studies.

a)

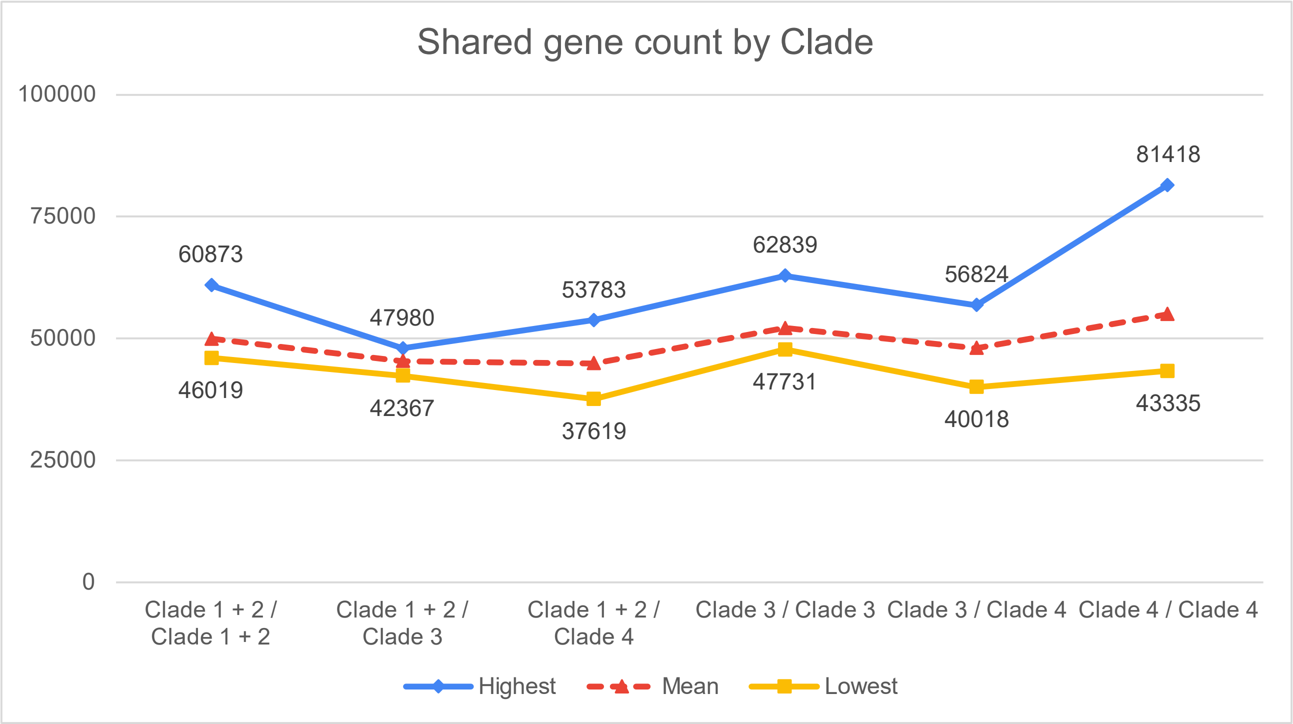

b)

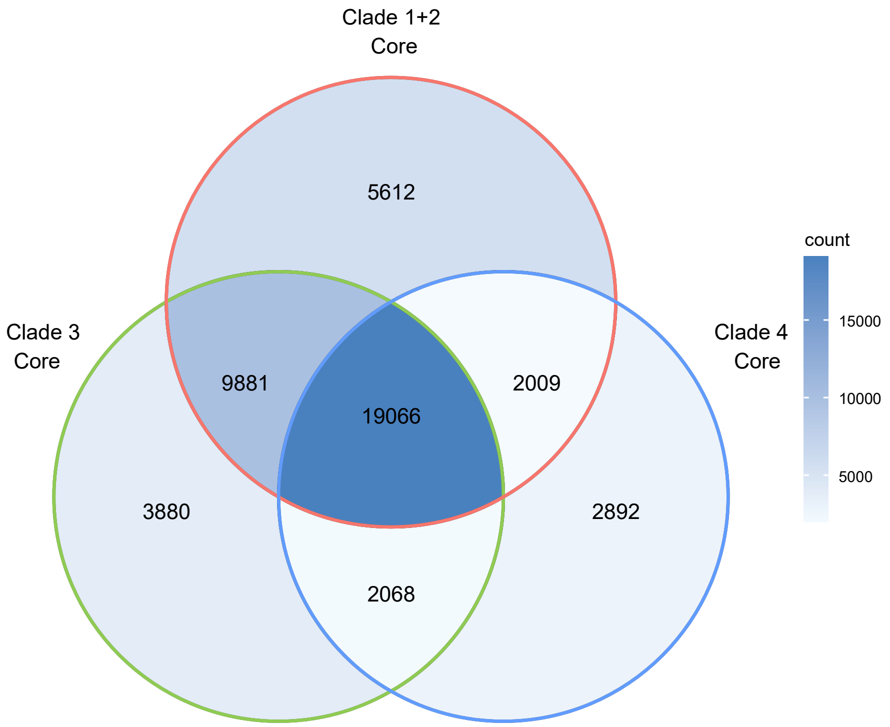

Fig. S10: Shared gene count among the three clades, Clade 1+2, Clade 3, and Clade 4. (a) Mean shared gene count among the three clades (b) number of core genes shared among the three clades

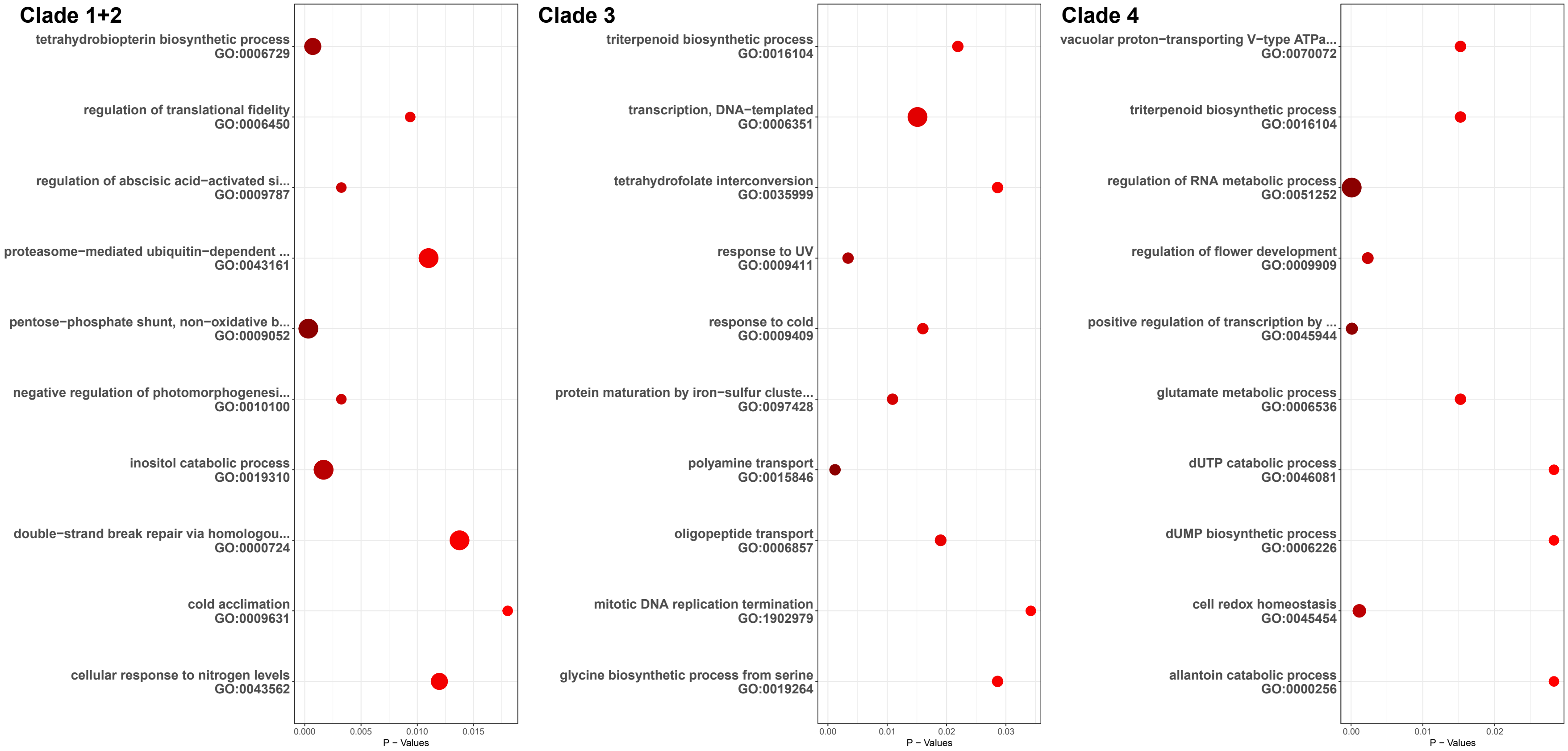

Fig. S11: Functional enrichment analysis of the unique core genome of Clade 1+2, Clade 3, and Clade 4 groups

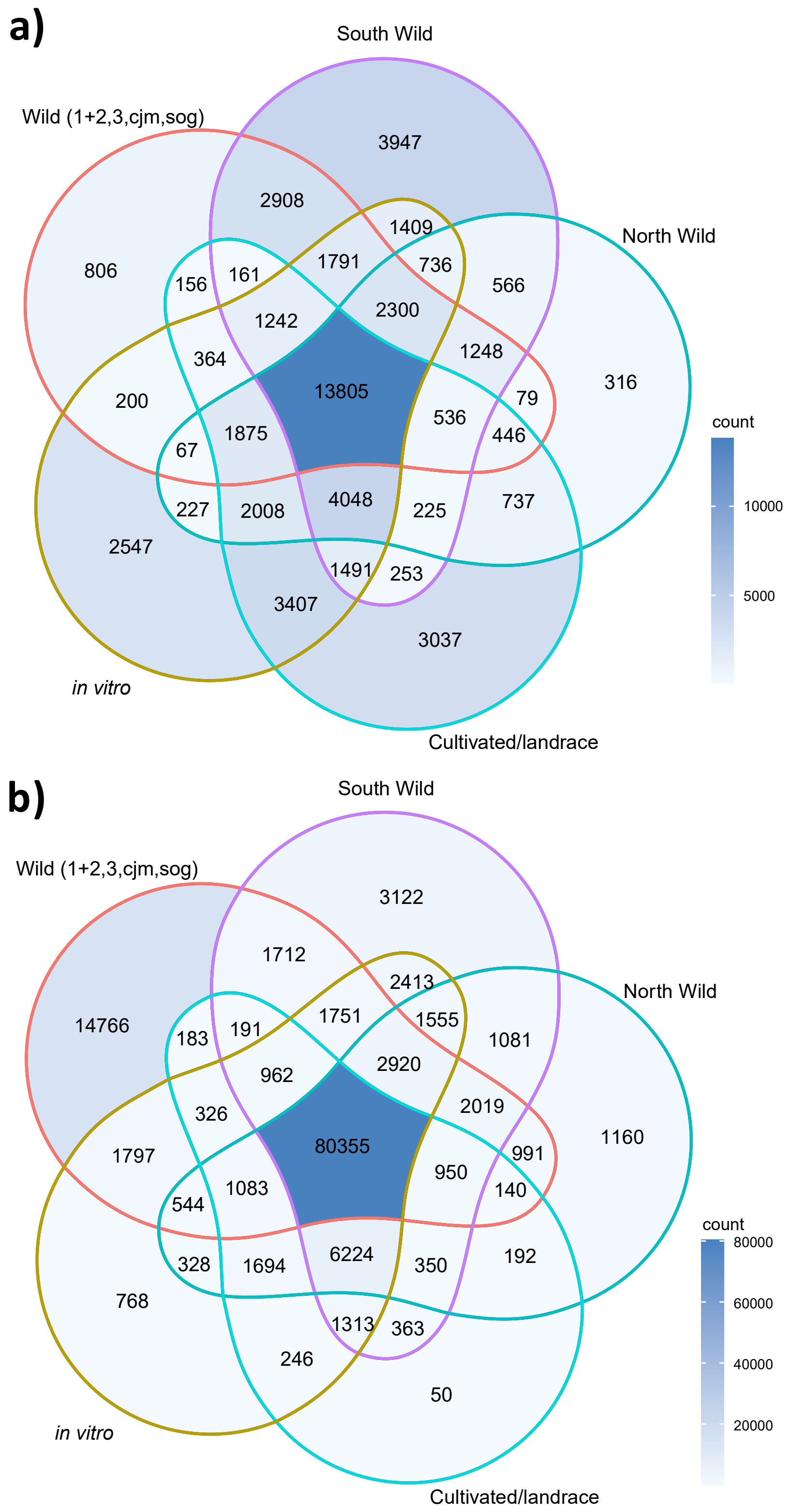

Fig. S12: Venn diagram of gene content among the different subgroups. a) Entire gene content in these subgroups and b) Core genes of the subgroups

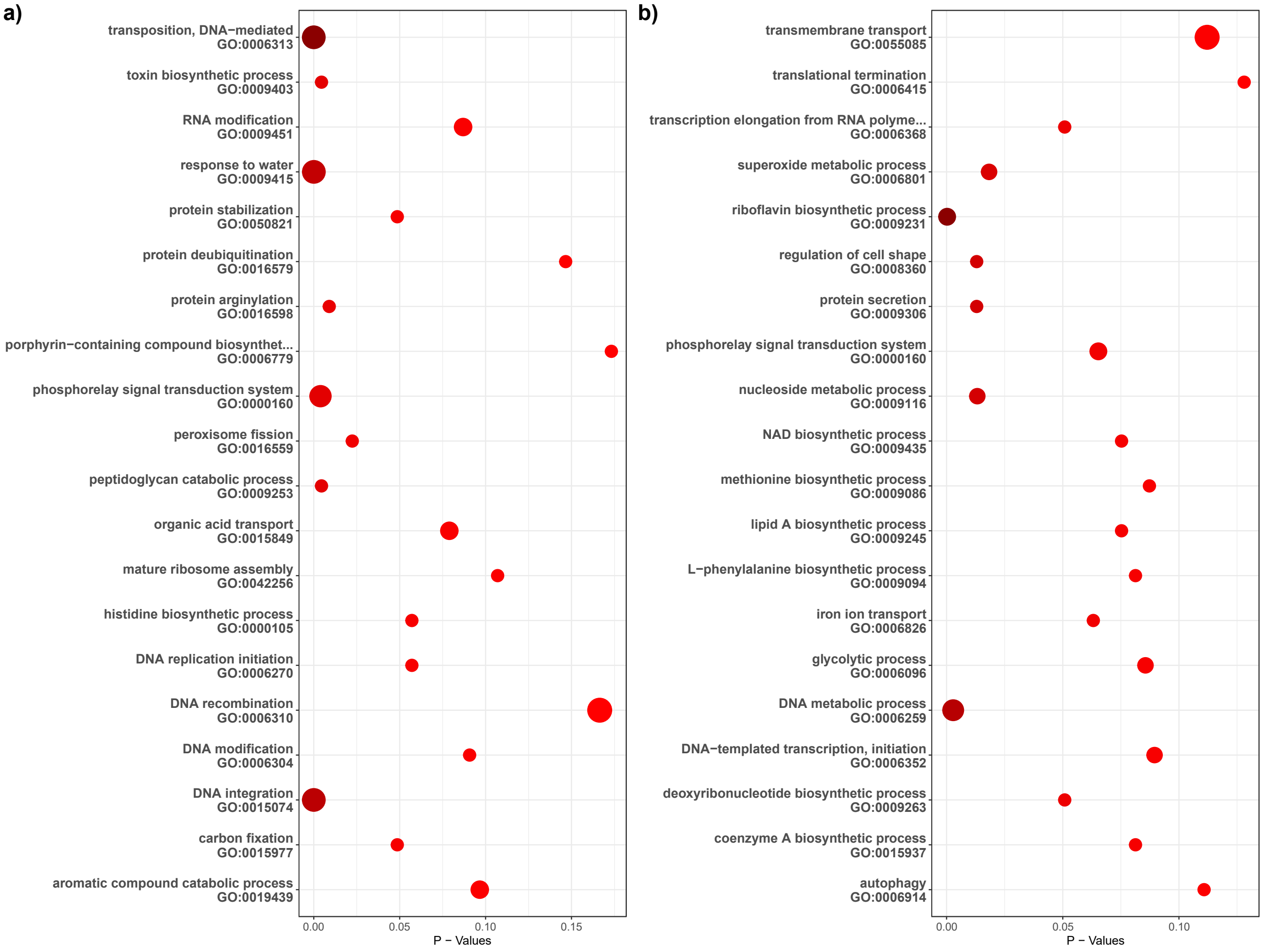

Fig. S13: Functional enrichment of (a) Genes present only in the *in vitro* subgroup (b) Genes present in all the remaining groups except the *in vitro* subgroup

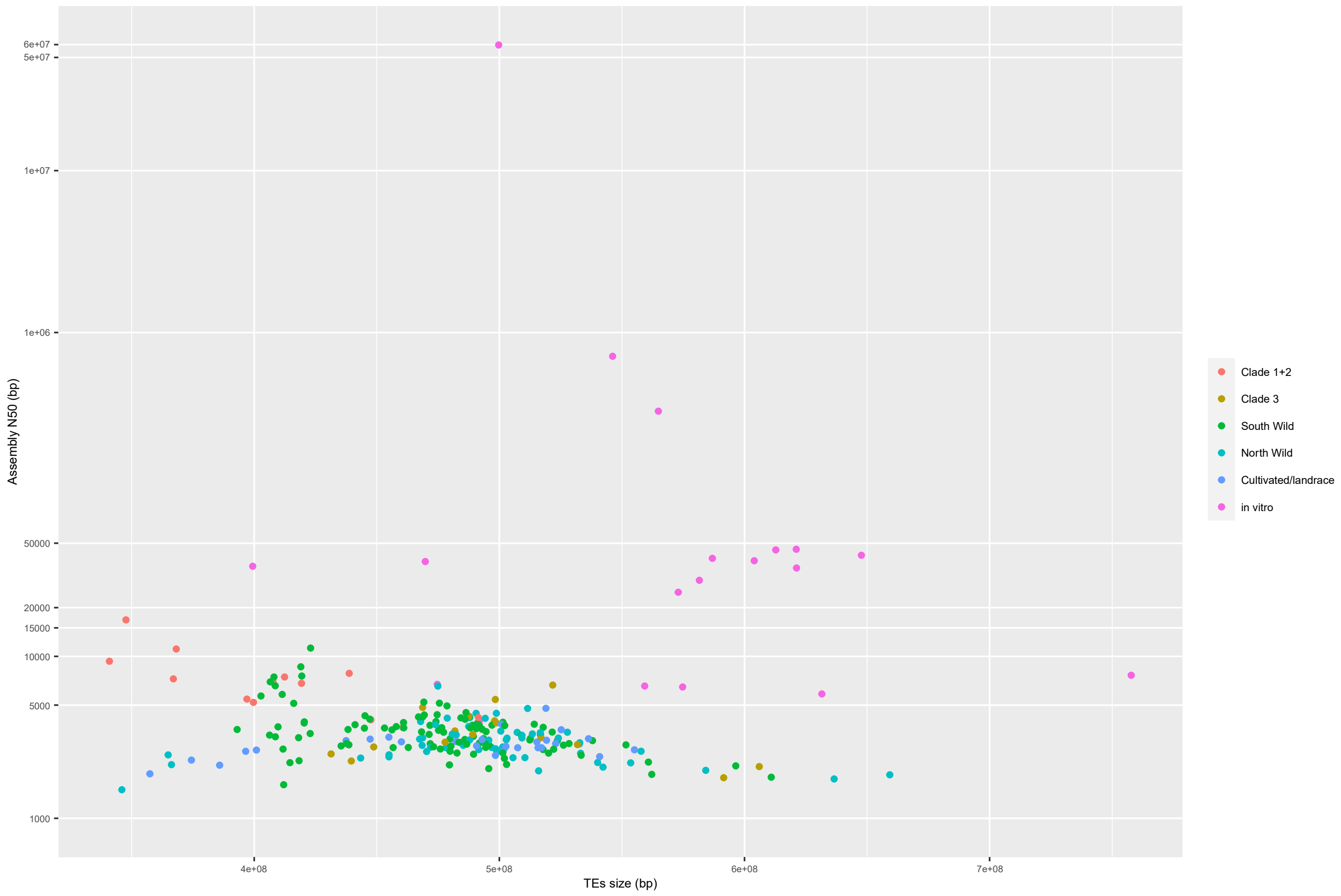

Fig. S14: A scatter plot of TEs size against the *de novo* assembly N50 size.
The possible effects of assembly contiguity on the TEs size are analyzed through this scatter plot. There is no correlation observed between the N50 value and the TEs size. In fact, the *in vitro* accession with the highest amount of TEs has lower N50 value than some of the Clade 1+2 accessions.

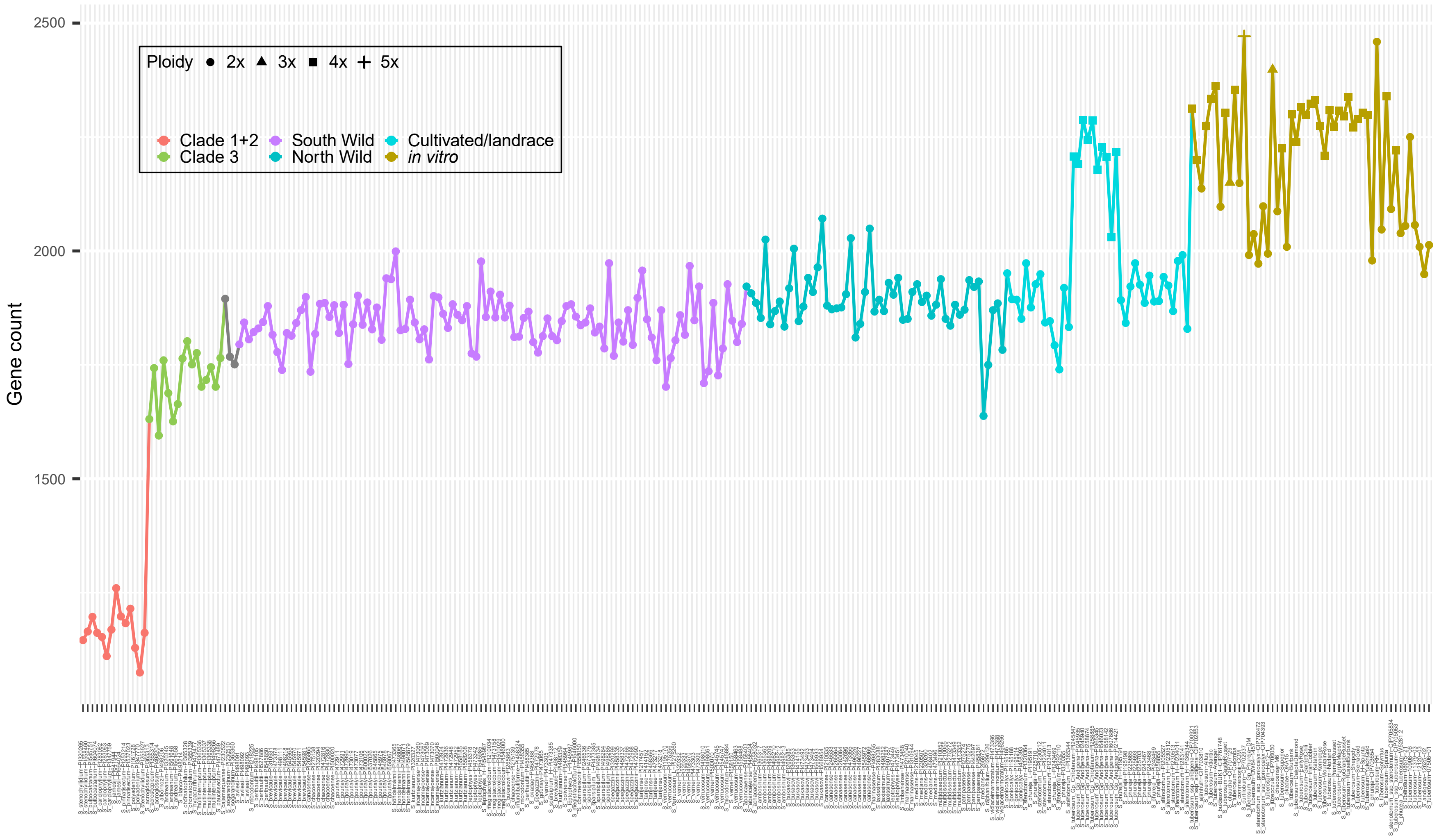

Fig. S15: Number of transposon related genes in each accession. The Clade 1+2 accessions have lower number of transposon genes compared to the rest, whereas the *in vitro* accessions have higher number of transposon genes.

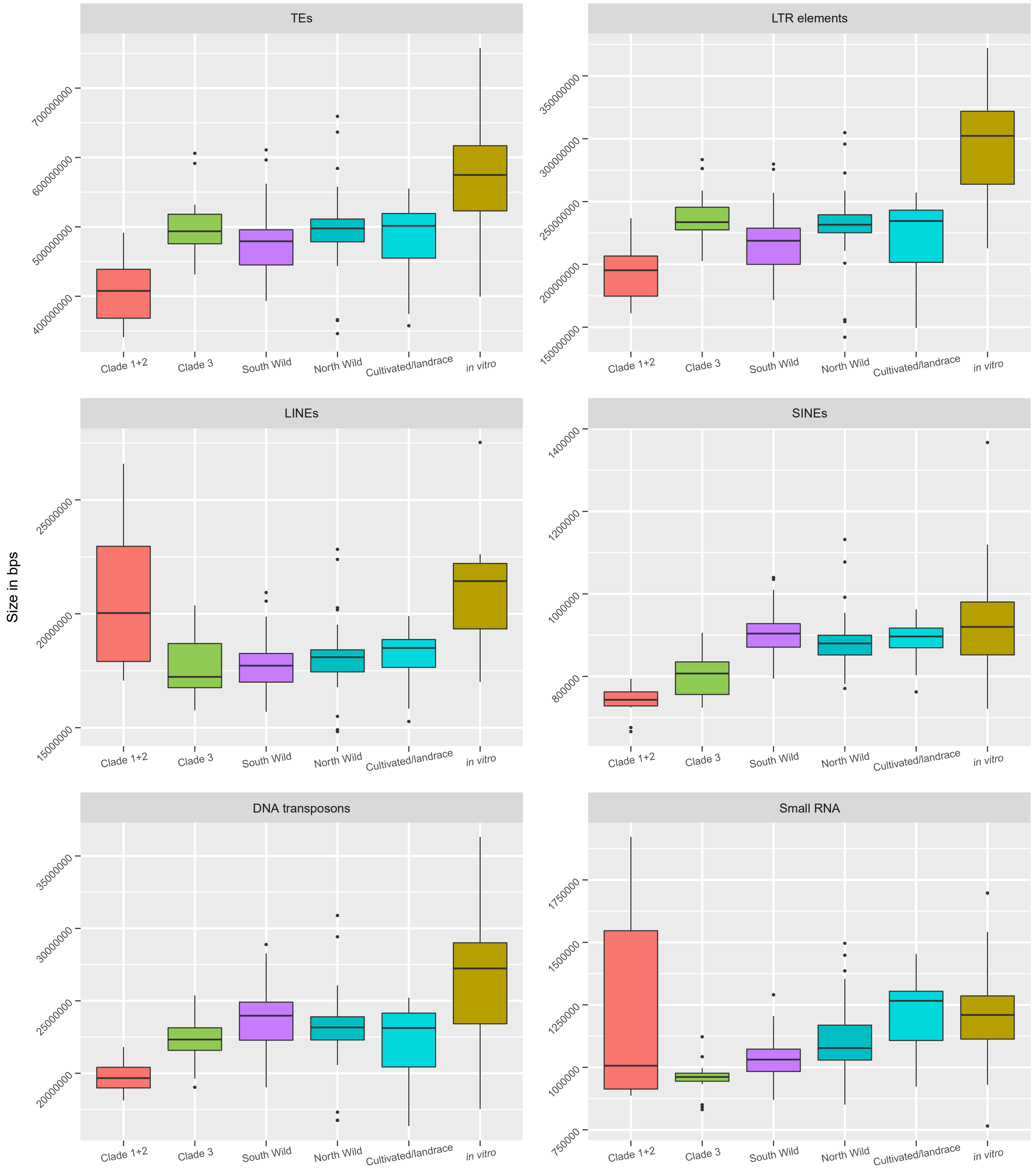

Fig. S16: Differences in major types of transposable elements in each specified group

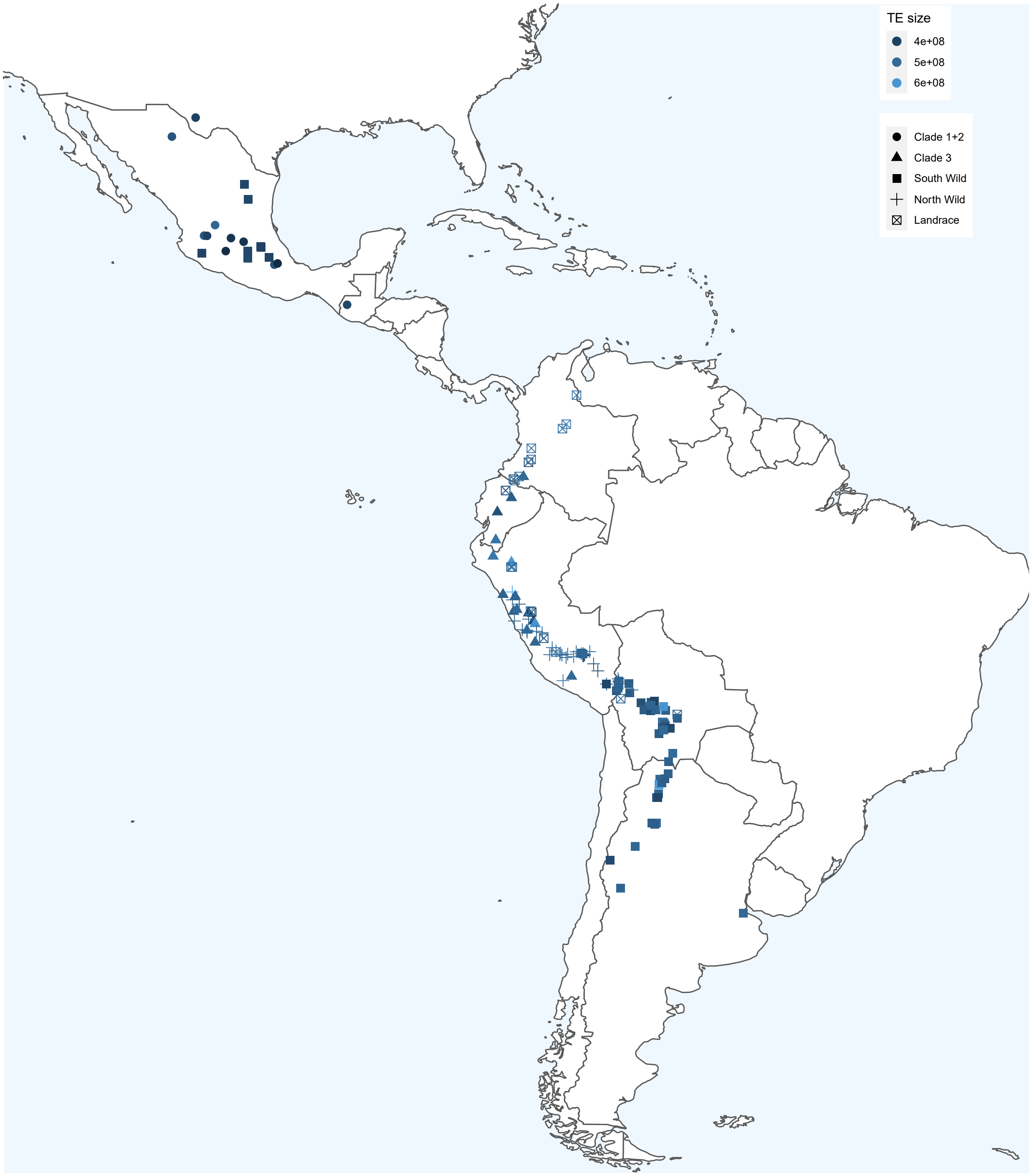

Fig. S17: Geographical distribution of the accessions from different groups, and their TEs size.

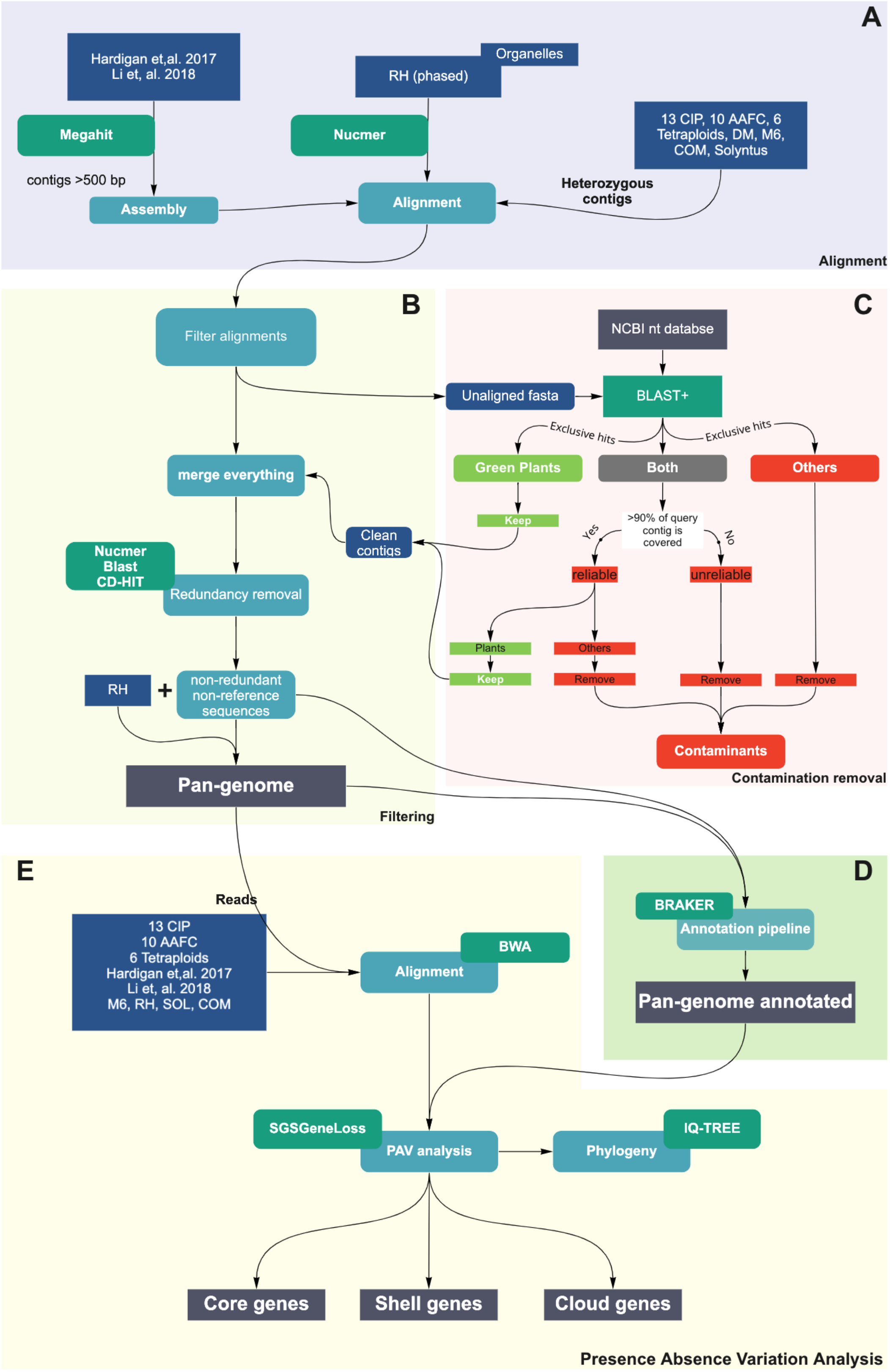

Fig. S18: Schematic representation of the pipeline used in construction of the *Solanum* section *Petota* pan-genome

Supplementary Tables

Table S1. Statistics of the *de novo* genome assembly of 296 accessions

Genome	Id	assembly (bp)	N50 (bp)	Gene count	Ploidy	Sequencing depth	Cultivated Status	PhyloClade	Subgroup
S. tuberosum (07506-01)	07506-01	900,692,377	35,151	63,570	2x	50	cultivar	Clade 4 north	*in vitro*
S. tuberosum (08675-21)	08675-21	916,666,787	45,878	65,810	2x	50	cultivar	Clade 4 north	*in vitro*
S. tuberosum (10908-06)	10908-06	714,258,418	6,719	72,182	2x	50	cultivar	Clade 4 north	*in vitro*
S. tuberosum (11379-03)	11379-03	952,662,949	42,127	65,655	2x	50	cultivar	Clade 4 north	*in vitro*
S. tuberosum (12120-03)	12120-03	845,658,231	29,502	63,926	2x	50	cultivar	Clade 4 north	*in vitro*
S. andigena (12625-02)	12625-02	829,063,217	24,896	63,265	2x	50	cultivar	Clade 4 north	*in vitro*
S. abancayense (PI 458403)	aba458403	763,981,864	2,756	64,096	2x	12	wild	Clade 4 north	North Wild
S. abancayense (PI 458404)	aba458404	770,265,347	2,367	63,529	2x	12	wild	Clade 4 north	North Wild
S. achacachense (PI 558032)	ach558032	741,331,819	4,187	66,082	2x	12	wild	Clade 4 north	North Wild
S. acroglossum (PI 365313)	acr365313	682,695,522	2,759	57,998	2x	12	wild	Clade 3	Clade 3
S. acroscopicum (PI 365314)	acr365314	749,356,496	3,998	62,839	2x	12	wild	Clade 3	Clade 3
S. acroglossum (PI 498204)	acr498204	662,987,248	2,498	57,440	2x	12	wild	Clade 3	Clade 3
S. tuberosum ssp. *andigena* (CIP 700921)	ADG1	841,416,273	121,761	74,982	4x	50	landrace	Clade 4 north	*in vitro*
S. tuberosum ssp. *andigena* (CIP 702853)	ADG2	991,109,043	4,745	72,741	4x	50	landrace	Clade 4 north	*in vitro*
S. ajanhuiri (CIP 703810)	AJH	828,902,572	6,563	70,504	2x	50	landrace	Clade 4 north	*in vitro*
S. albornozii (PI 498206)	alb498206	761,342,435	6,647	61,040	2x	12	wild	Clade 3	Clade 3
S. tuberosum (Altus)	Altus	2,327,702,221	1,925,378	75,200	4x	Hybrid	cultivar	Clade 4 north	*in vitro*
S. ambosinum (PI 365317)	amb365317	746,610,816	2,685	63,042	2x	12	wild	Clade 4 north	North Wild
S. ambosinum (PI 365362)	amb365362	990,827,380	1,856	68,045	2x	12	wild	Clade 4 north	North Wild
S. ambosinum (PI 498209)	amb498209	776,081,986	3,136	62,928	2x	12	wild	Clade 4 north	North Wild
S. ambosinum (PI 498210)	amb498210	767,445,345	3,058	63,205	2x	12	wild	Clade 4 north	North Wild
S. ambosinum (PI 498212)	amb498212	754,976,950	2,742	62,780	2x	12	wild	Clade 4 north	North Wild
S. ambosinum (PI 498213)	amb498213	744,968,163	4,449	62,118	2x	12	wild	Clade 4 north	North Wild
S. andreanum (PI 320345)	and320345	737,114,745	5,419	61,788	2x	12	wild	Clade 3	Clade 3
S. andreanum (PI 561648)	and561648	697,828,667	4,837	59,683	2x	12	wild	Clade 3	Clade 3
S. andreanum (PI 561658)	and561658	673,568,374	2,259	59,235	2x	12	wild	Clade 3	Clade 3

S. tuberosum (Atlantic)	Atlantic	2,717,416,440	36,909,319	76,777	4x	Hybrid	cultivar	Clade 4 north	*in vitro*
S. tuberosum (Avenger)	Avenger	2,606,102,302	1,580,890	76,995	4x	Hybrid	cultivar	Clade 4 north	*in vitro*
S. avilesii (PI 498091)	avi498091	713,879,527	3,517	63,382	2x	12	wild	Clade 4 south	South Wild
S. avilesii (PI 498092)	avi498092	743,374,148	3,095	64,124	2x	12	wild	Clade 4 south	South Wild
S. avilesii (PI 498093)	avi498093	734,796,172	2,888	63,279	2x	12	wild	Clade 4 south	South Wild
S. microdontum (PI 218225)	ber218225	741,323,676	3,400	64,343	2x	12	wild	Clade 4 south	South Wild
S. berthaultii (PI 498105)	ber498105	749,098,464	3,756	64,348	2x	12	wild	Clade 4 south	South Wild
S. berthaultii (PI 527886)	ber527886	766,655,755	3,919	65,514	2x	12	wild	Clade 4 south	South Wild
S. berthaultii (PI 545850)	ber545850	789,952,173	3,278	65,569	2x	12	wild	Clade 4 south	South Wild
S. blanco (PI 498214)	bla498214	721,284,445	3,463	60,480	2x	12	wild	Clade 3	Clade 3
S. stenophyllidium (PI 320265)	bra320265	697,919,867	4,059	55,547	2x	12	wild	Clade 1+2	Clade 1+2
S. stenophyllidium (PI 558460)	bra558460	702,815,315	3,640	56,287	2x	12	wild	Clade 1+2	Clade 1+2
S. brevicaule (PI 310931)	bre310931	739,054,457	3,629	64,081	2x	12	wild	Clade 4 south	South Wild
S. brevicaule (PI 473378)	bre473378	715,638,958	3,684	63,160	2x	12	wild	Clade 4 south	South Wild
S. brevicaule (PI 498111)	bre498111	689,063,797	2,843	61,597	2x	12	wild	Clade 4 south	South Wild
S. brevicaule (PI 498218)	bre498218	732,378,826	3,297	64,128	2x	12	wild	Clade 4 south	South Wild
S. brevicaule (PI 545968)	bre545968	766,613,458	2,832	63,912	2x	12	wild	Clade 4 south	South Wild
S. brevicaule (PI 545970)	bre545970	777,013,150	2,151	64,113	2x	12	wild	Clade 4 south	South Wild
S. brevicaule (PI 545971)	bre545971	800,856,697	3,017	65,634	2x	12	wild	Clade 4 south	South Wild
S. brevicaule (PI 545981)	bre545981	851,031,193	1,868	66,824	2x	12	wild	Clade 4 south	South Wild
S. bukasovii (CIP 761748)	BUK1	1,144,308,148	7,644	69,626	2x	50	wild	Clade 4 north	*in vitro*
S. bukasovii (CIP 761748)	BUK2	615,681,290	1,872,399	65,120	2x	50	wild	NA	NA
S. bukasovii (PI 266385)	buk266385	761,212,581	2,584	65,212	2x	12	wild	Clade 4 north	North Wild
S. bukasovii (PI 365353)	buk365353	785,834,405	3,045	63,277	2x	12	wild	Clade 4 north	North Wild
S. bukasovii (PI 414155)	buk414155	763,905,760	2,698	62,754	2x	12	wild	Clade 4 north	North Wild
S. bukasovii (PI 473492)	buk473492	761,058,704	3,394	63,378	2x	12	wild	Clade 4 north	North Wild
S. bukasovii (PI 473493)	buk473493	792,905,319	3,131	65,722	2x	12	wild	Clade 4 north	North Wild
S. bukasovii (PI 473494)	buk473494	765,956,392	3,126	65,009	2x	12	wild	Clade 4 north	North Wild
S. bukasovii (PI 568933)	buk568933	818,826,041	2,070	64,371	2x	12	wild	Clade 4 north	North Wild
S. bukasovii (PI 568954)	buk568954	877,219,859	1,977	67,460	2x	12	wild	Clade 4 north	North Wild
S. bulbocastanum (PI 545751)	bul545751	646,423,176	5,198	54,960	2x	12	wild	Clade 1+2	Clade 1+2
S. bulbocastanum (PI 604074)	bul604074	653,812,958	7,459	56,488	2x	12	wild	Clade 1+2	Clade 1+2
S. cajamarquense (PI 230522)	caj230522	828,122,061	2,116	64,332	2x	12	wild	NA	NA
S. canasense (PI 210035)	can210035	743,190,412	3,693	63,503	2x	12	wild	Clade 4 north	North Wild
S. canasense (PI 246533)	can246533	751,403,827	2,657	62,707	2x	12	wild	Clade 4 north	North Wild
S. canasense (PI 265864)	can265864	734,230,376	2,729	62,887	2x	12	wild	Clade 4 north	North Wild

S. canasense (PI 265865)	can265865	689,466,627	3,786	63,187	2x	12	wild	Clade 4 south	South Wild
S. canasense (PI 283084)	can283084	763,585,281	2,603	63,073	2x	12	wild	Clade 4 north	North Wild
S. canasense (PI 442696)	can442696	811,179,730	2,521	64,421	2x	12	wild	Clade 4 north	North Wild
S. canasense (PI 473355)	can473355	840,987,445	2,595	66,700	2x	12	wild	Clade 4 north	North Wild
S. canasense (PI 498226)	can498226	719,316,061	3,140	61,789	2x	12	wild	Clade 4 north	North Wild
S. canasense (PI 498227)	can498227	715,472,643	3,958	63,033	2x	12	wild	Clade 4 north	North Wild
S. canasense (PI 545972)	can545972	734,614,972	4,156	64,936	2x	12	wild	Clade 4 north	North Wild
S. canasense (PI 568969)	can568969	849,450,184	2,200	64,772	2x	12	wild	Clade 4 north	North Wild
S. cardiophyllum (PI 283062)	car283062	659,018,264	6,815	57,408	2x	12	wild	Clade 1+2	Clade 1+2
S. cardiophyllum (PI 283063)	car283063	643,242,320	7,013	56,855	2x	12	wild	Clade 1+2	Clade 1+2
S. cardiophyllum (PI 347759)	car347759	689,165,664	7,857	58,097	2x	12	wild	Clade 1+2	Clade 1+2
S. tuberosum (CastleRusset)	Castle Russet	2,529,459,982	25,966,585	75,849	4x	Hybrid	cultivar	Clade 4 north	*in vitro*
S. chaucha (CIP 707129)	CHA	790,336,793	4,840	69,893	3x	50	landrace	Clade 4 north	*in vitro*
S. chacoense (PI 275138)	cha275138	782,844,170	3,113	63,006	2x	12	wild	Clade 4 south	South Wild
S. chacoense (PI 320294)	cha320294	759,674,270	3,761	64,266	2x	12	wild	Clade 4 south	South Wild
S. chacoense (PI 472816)	cha472816	782,705,961	3,046	64,071	2x	12	wild	Clade 4 south	South Wild
S. chacoense (PI 472830)	cha472830	784,974,511	3,811	64,524	2x	12	wild	Clade 4 south	South Wild
S. chacoense (PI 500020)	cha500020	803,464,895	2,829	64,518	2x	12	wild	Clade 4 south	South Wild
S. chomatophilum (PI 365328)	cho365328	886,268,572	2,087	62,393	2x	12	wild	Clade 3	Clade 3
S. chomatophilum (PI 365339)	cho365339	869,956,934	1,781	61,056	2x	12	wild	Clade 3	Clade 3
S. tuberosum (Colomba)	Colomba	2,180,359,322	1,208,878	75,683	4x	Hybrid	cultivar	Clade 4 north	*in vitro*
S. commersonii (COM)	COM	729,602,650	38,519	74,981	2x	Hybrid	wild	Clade 4 south	*in vitro*
S. curtilobum (CIP 702937)	CUR	1,207,837,434	3,247	81,246	5x	50	landrace	Clade 4 north	*in vitro*
S. tuberosum Gp. *Phureja* (DM)	DM	741,585,035	59,670,755	67,318	2x	Hybrid	cultivar	Clade 4 north	*in vitro*
S. tuberosum (DW84-1457)	DW84-1457	885,322,277	38,963	65,709	2x	50	cultivar	Clade 4 north	*in vitro*
S. stenotomum ssp. *goniocalyx* (CIP 702472)	GON1	855,795,280	326,785	65,269	2x	Hybrid	landrace	Clade 4 north	*in vitro*
S. goniocalyx (PI 195186)	gon195186	802,478,196	2,872	63,913	2x	12	landrace	Clade 4 north	Cultivated/landrace
S. goniocalyx (PI 195188)	gon195188	786,808,201	4,777	61,803	2x	12	landrace	Clade 4 north	Cultivated/landrace
S. goniocalyx (PI 195214)	gon195214	763,678,654	2,590	62,153	2x	12	landrace	Clade 4 north	Cultivated/landrace
S. stenotomum ssp. *goniocalyx* (CIP 704393)	GON2	770,898,988	3,968	69,041	2x	50	landrace	Clade 4 north	*in vitro*
S. goniocalyx (PI 458393)	gon458393	757,613,141	2,445	61,658	2x	12	landrace	Clade 4 north	Cultivated/landrace
S. gourlayi (PI 472911)	gou472911	762,291,778	2,741	64,620	2x	12	wild	Clade 4 south	South Wild
S. gourlayi (PI 472991)	gou472991	648,309,057	1,612	65,576	2x	12	wild	Clade 4 south	South Wild
S. gourlayi (PI 472995)	gou472995	708,198,932	2,733	63,085	2x	12	wild	Clade 4 south	South Wild

S. gourlayi (PI 473019)	gou473019	792,895,316	3,408	65,949	2x	12	wild	Clade 4 south	South Wild
S. gourlayi (PI 473077)	gou473077	763,247,505	3,207	64,333	2x	12	wild	Clade 4 south	South Wild
S. gourlayi (PI 473106)	gou473106	784,381,528	2,659	64,829	2x	12	wild	Clade 4 south	South Wild
S. gourlayi (PI 500022)	gou500022	764,519,180	2,027	65,787	2x	12	wild	Clade 4 south	South Wild
S. gourlayi (PI 537026)	gou537026	844,883,231	2,224	64,688	2x	12	wild	Clade 4 south	South Wild
S. gourlayi (PI 545865)	gou545865	815,778,272	2,451	65,344	2x	12	wild	Clade 4 south	South Wild
S. gourlayi (PI 545975)	gou545975	758,929,143	2,920	63,874	2x	12	wild	Clade 4 south	South Wild
S. gourlayi (PI 545978)	gou545978	796,801,554	2,527	65,173	2x	12	wild	Clade 4 south	South Wild
S. gourlayi (PI 558067)	gou558067	911,926,984	1,794	67,213	2x	12	wild	Clade 4 south	South Wild
S. tuberosum (H412-1)	H412-1	905,005,116	45,444	64,821	2x	50	cultivar	Clade 4 north	*in vitro*
S. stenotomum (PI 320364)	haw320364	803,797,200	2,889	63,532	2x	12	landrace	Clade 4 north	Cultivated/landrace
S. hondelmannii (PI 473365)	hon473365	823,317,929	3,018	67,062	2x	12	wild	Clade 4 south	South Wild
S. hondelmannii (PI 498067)	hon498067	748,209,453	2,530	63,194	2x	12	wild	Clade 4 south	South Wild
S. hondelmannii (PI 498071)	hon498071	687,536,877	3,536	62,512	2x	12	wild	Clade 4 south	South Wild
S. hondelmannii (PI 545879)	hon545879	742,289,363	2,679	64,782	2x	12	wild	Clade 4 south	South Wild
S. kurtzianum (PI 320327)	hua320327	736,878,242	3,956	64,706	2x	12	wild	Clade 4 south	South Wild
S. marinasense (PI 498255)	hua498255	739,046,686	2,824	62,347	2x	12	wild	Clade 4 north	North Wild
S. hypacrarthrum (PI 473477)	hyp473477	775,444,979	3,147	62,123	2x	12	wild	Clade 3	Clade 3
S. incamayoense (PI 473060)	inc473060	734,212,607	3,495	63,782	2x	12	wild	Clade 4 south	South Wild
S. incamayoense (PI 473067)	inc473067	749,347,451	4,251	65,003	2x	12	wild	Clade 4 south	South Wild
S. incamayoense (PI 473069)	inc473069	697,098,638	3,612	62,387	2x	12	wild	Clade 4 south	South Wild
S. incamayoense (PI 473070)	inc473070	753,145,111	4,010	64,754	2x	12	wild	Clade 4 south	South Wild
S. incamayoense (PI 500048)	inc500048	752,149,892	2,488	63,628	2x	12	wild	Clade 4 south	South Wild
S. jamesii (PI 641944)	jam641944	627,934,303	9,565	60,873	2x	12	wild	Clade 1+2	Clade 1+2
S. jamesii (PI 664024)	jam664024	630,306,268	5,448	59,262	2x	12	wild	Clade 1+2	Clade 1+2
S. juzepczukii (CIP 706050)	JUZ	1,002,422,951	7,414	81,418	3x	50	landrace	Clade 4 north	*in vitro*
S. kurtzianum (PI 472924)	kur472924	840,277,395	2,840	65,153	2x	12	wild	Clade 4 south	South Wild
S. kurtzianum (PI 472936)	kur472936	778,691,210	3,262	64,144	2x	12	wild	Clade 4 south	South Wild
S. kurtzianum (PI 472948)	kur472948	785,687,374	3,648	64,663	2x	12	wild	Clade 4 south	South Wild
S. kurtzianum (PI 472952)	kur472952	804,147,853	2,894	64,479	2x	12	wild	Clade 4 south	South Wild
S. kurtzianum (PI 558185)	kur558185	744,529,561	3,067	64,058	2x	12	wild	Clade 4 south	South Wild
S. kurtzianum (PI 558208)	kur558208	752,843,197	3,738	64,549	2x	12	wild	Clade 4 south	South Wild
S. laxissimum (PI 283088)	lax283088	735,113,927	3,274	63,367	2x	12	wild	Clade 4 north	North Wild
S. laxissimum (PI 498252)	lax498252	721,059,959	6,551	64,748	2x	12	wild	Clade 4 north	North Wild
S. laxissimum (PI 607887)	lax607887	778,750,244	4,771	66,670	2x	12	wild	Clade 4 north	North Wild
S. leptophyes (PI 458378)	lep458378	715,749,748	2,738	62,028	2x	12	wild	Clade 4 south	South Wild

S. leptophyes (PI 473342)	lep473342	715,444,523	3,620	63,220	2x	12	wild	Clade 4 south	South Wild
S. leptophyes (PI 473446)	lep473446	797,698,918	3,400	64,416	2x	12	wild	Clade 4 north	North Wild
S. leptophyes (PI 473451)	lep473451	807,221,082	2,927	65,479	2x	12	wild	Clade 4 north	North Wild
S. leptophyes (PI 545985)	lep545985	732,182,385	3,418	64,233	2x	12	wild	Clade 4 south	South Wild
S. leptophyes (PI 545987)	lep545987	756,022,474	3,582	64,676	2x	12	wild	Clade 4 south	South Wild
S. limbaniense (PI 473468)	lim473468	777,994,913	3,320	63,197	2x	12	wild	Clade 4 north	North Wild
S. chacoense (M6)	M6	825,767,562	713,601	72,048	2x	Hybrid	wild	Clade 4 south	*in vitro*
S. marinasense (PI 210040)	mar210040	700,208,060	2,468	61,948	2x	12	wild	Clade 4 north	North Wild
S. marinasense (PI 310944)	mar310944	775,683,337	2,369	64,089	2x	12	wild	Clade 4 north	North Wild
S. medians (PI 210045)	med210045	783,629,910	1,964	65,963	2x	12	wild	Clade 4 north	North Wild
S. medians (PI 230507)	med230507	745,497,706	3,049	65,111	2x	12	wild	Clade 4 north	North Wild
S. medians (PI 320260)	med320260	752,740,068	4,145	65,567	2x	12	wild	Clade 4 north	North Wild
S. medians (PI 458402)	med458402	752,399,152	3,041	64,762	2x	12	wild	Clade 4 north	North Wild
S. medians (PI 473496)	med473496	734,911,193	3,309	65,182	2x	12	wild	Clade 4 north	North Wild
S. megistacrolobum (PI 210034)	meg210034	728,065,524	4,347	65,424	2x	12	wild	Clade 4 south	South Wild
S. megistacrolobum (PI 473158)	meg473158	754,628,320	3,104	66,137	2x	12	wild	Clade 4 south	South Wild
S. megistacrolobum (PI 500029)	meg500029	733,424,540	4,945	66,210	2x	12	wild	Clade 4 south	South Wild
S. megistacrolobum (PI 546000)	meg546000	759,349,569	3,431	66,465	2x	12	wild	Clade 4 south	South Wild
S. multiinterruptum (PI 210044)	mul210044	759,774,678	3,455	NA	2x	12	wild/landrace hybrid	NA	NA
S. multidissectum (PI 210052)	mul210052	811,830,842	2,210	65,503	2x	12	wild	Clade 4 north	North Wild
S. multidissectum (PI 210055)	mul210055	715,946,344	3,085	63,972	2x	12	wild	Clade 4 north	North Wild
S. multidissectum (PI 275272)	mul275272	721,806,282	2,819	63,136	2x	12	wild	Clade 4 north	North Wild
S. multiinterruptum (PI 365336)	mul365336	726,768,512	2,951	61,142	2x	12	wild	Clade 3	Clade 3
S. multiinterruptum (PI 365337)	mul365337	738,128,703	3,296	61,976	2x	12	wild	Clade 3	Clade 3
S. multiinterruptum (PI 365338)	mul365338	750,280,579	3,888	61,872	2x	12	wild	Clade 3	Clade 3
S. multidissectum (PI 473349)	mul473349	723,825,954	2,585	64,246	2x	12	wild	Clade 4 north	North Wild
S. multidissectum (PI 473352)	mul473352	688,288,023	2,356	64,650	2x	12	wild	Clade 4 north	North Wild
S. multiinterruptum (PI 498266)	mul498266	734,432,269	4,244	61,952	2x	12	wild	Clade 3	Clade 3
S. tuberosum (Superior)	NPV_FT	1,251,939,777	1,575	72,109	4x	16	cultivar	Clade 4 north	*in vitro*
S. okadae (OKA15)	OKA15	617,821,277	36,031	67,242	2x	50	wild	Clade 4 south	*in vitro*
S. pampasense (PI 275274)	pam275274	722,142,785	2,749	63,577	2x	12	wild	Clade 4 north	North Wild
S. pampasense (PI 275275)	pam275275	754,892,637	4,452	65,326	2x	12	wild	Clade 4 north	North Wild
S. pampasense (PI 442697)	pam442697	780,359,808	3,380	64,027	2x	12	wild	Clade 4 north	North Wild
S. pampasense (PI 458381)	pam458381	754,340,467	3,038	64,540	2x	12	wild	Clade 4 north	North Wild
S. paucissectum (PI 473489)	pau473489	787,404,001	2,852	61,973	2x	12	wild	Clade 3	Clade 3

S. phureja (PI 195191)	PDP_AA	646,335,849	2,639	60,135	2x	8	landrace	Clade 4 north	Cultivated/landrace
S. stenotomum (PI 195204)	PDP_AB	585,496,789	1,501	NA	2x	8	wild/landrace hybrid	NA	NA
S. multidissectum (PI 210044)	PDP_AC	603,301,651	2,145	NA	2x	8	wild/landrace hybrid	NA	NA
S. phureja (PI 225710)	PDP_AD	690,316,508	3,018	61,150	2x	8	landrace	Clade 4 north	Cultivated/landrace
S. stenotomum (PI 230512)	PDP_AE	725,224,780	2,967	63,749	2x	8	landrace	Clade 4 north	Cultivated/landrace
S. stenotomum (PI 234011)	PDP_AF	639,469,147	2,593	60,411	2x	8	landrace	Clade 4 north	Cultivated/landrace
S. stenotomum (PI 234013)	PDP_AG	628,893,590	2,128	60,298	2x	8	landrace	Clade 4 north	Cultivated/landrace
S. phureja (PI 243469)	PDP_AH	591,198,508	1,883	57,180	2x	8	landrace	Clade 4 north	Cultivated/landrace
S. bukasovii (PI 265863)	PDP_AI	674,603,083	2,206	64,731	2x	8	wild	Clade 4 south	South Wild
S. medians (PI 265872)	PDP_AJ	494,037,684	1,393	55,962	2x	8	wild	Clade 4 north	North Wild
S. chacoense (PI 275139)	PDP_AK	662,258,341	2,676	62,238	2x	8	wild	Clade 4 south	South Wild
S. stenotomum (PI 292110)	PDP_AL	612,646,683	2,289	57,820	2x	8	landrace	Clade 4 north	Cultivated/landrace
S. raphanifolium (PI 296126)	PDP_AM	602,468,227	2,463	62,025	2x	8	wild	Clade 4 north	North Wild
S. phureja (PI 320355)	PDP_AN	715,512,076	3,170	61,080	2x	8	landrace	Clade 4 north	Cultivated/landrace
S. stenotomum (PI 365344)	PDP_AO	703,940,204	3,083	61,679	2x	8	landrace	Clade 4 north	Cultivated/landrace
S. infundibuliforme (PI 458324)	PDP_AP	655,068,989	3,186	63,141	2x	8	wild	Clade 4 south	South Wild
S. microdontum (PI 458355)	PDP_AQ	674,973,357	3,146	64,407	2x	8	wild	Clade 4 south	South Wild
S. berthaultii (PI 458365)	PDP_AR	701,958,970	3,603	65,047	2x	8	wild	Clade 4 south	South Wild
S. okadae (PI 458368)	PDP_AS	635,023,887	3,535	63,100	2x	8	wild	Clade 4 south	South Wild
S. spegazzinii (PI 472978)	PDP_AT	665,592,962	3,943	62,665	2x	8	wild	Clade 4 south	South Wild
S. gourlayi (PI 473065)	PDP_AU	697,768,446	2,895	63,857	2x	8	wild	Clade 4 south	South Wild
S. vernei (PI 473305)	PDP_AV	657,613,773	3,671	63,080	2x	8	wild	Clade 4 south	South Wild
S. sparsipilum (PI 473385)	PDP_AW	688,853,193	2,268	64,649	2x	8	wild	Clade 4 south	South Wild
S. brevicaule (PI 498112)	PDP_AX	661,040,436	3,264	63,925	2x	8	wild	Clade 4 south	South Wild
S. kurtzianum (PI 498359)	PDP_AY	671,935,619	3,338	62,985	2x	8	wild	Clade 4 south	South Wild
S. boliviense (PI 545964)	PDP_AZ	701,243,020	4,298	66,584	2x	8	wild	Clade 4 south	South Wild
S. leptophyes (PI 545987)	PDP_BA	699,746,749	2,798	64,520	2x	8	wild	Clade 4 south	South Wild
S. megistracrolobum (PI 546000)	PDP_BB	669,095,480	3,877	65,104	2x	8	wild	Clade 4 south	South Wild
S. commersonii (PI 558050)	PDP_BC	721,076,492	4,228	65,220	2x	8	wild	Clade 4 south	South Wild
S. tuberosum Gp. *Chilotanum* (PI 245847)	PDP_BD	909,095,412	1,919	71,492	4x	16	landrace	Clade 4 north	Cultivated/landrace
S. tuberosum Gp. *Andigena* (PI 245935)	PDP_BE	909,764,440	2,252	70,918	4x	16	landrace	Clade 4 north	Cultivated/landrace
S. tuberosum Gp. *Andigena* (PI 245940)	PDP_BF	952,935,710	1,699	73,594	4x	16	landrace	Clade 4 north	Cultivated/landrace

S. tuberosum Gp. *Andigena* (PI 258874)	PDP_BG	959,170,054	1,708	73,356	4x	16	landrace	Clade 4 north	Cultivated/landrace
S. tuberosum Gp. *Chilotanum* (PI 258885)	PDP_BH	975,764,833	1,651	73,136	4x	16	landrace	Clade 4 north	Cultivated/landrace
S. tuberosum Gp. *Andigena* (PI 365345)	PDP_BI	909,994,079	2,445	71,073	4x	16	landrace	Clade 4 north	Cultivated/landrace
S. tuberosum Gp. *Andigena* (PI 546023)	PDP_BJ	892,102,292	1,914	71,678	4x	16	landrace	Clade 4 north	Cultivated/landrace
S. tuberosum Gp. *Andigena* (PI 558142)	PDP_BK	887,343,790	2,288	71,377	4x	16	landrace	Clade 4 north	Cultivated/landrace
S. tuberosum Gp. *Andigena* (PI 607886)	PDP_BL	773,006,064	2,847	68,183	4x	16	landrace	Clade 4 north	Cultivated/landrace
S. tuberosum (Burbank)	PDP_BM	913,234,446	1,691	73,524	4x	16	cultivar	Clade 4 north	*in vitro*
S. tuberosum (DakotaDiamond)	PDP_BN	905,978,287	1,666	72,678	4x	16	cultivar	Clade 4 north	*in vitro*
S. tuberosum (EarlyRose)	PDP_BO	1,008,389,525	1,628	74,517	4x	16	cultivar	Clade 4 north	*in vitro*
S. tuberosum (GarnetChili)	PDP_BP	1,120,202,931	1,521	75,660	4x	16	cultivar	Clade 4 north	*in vitro*
S. tuberosum (IrishCobbler)	PDP_BQ	1,143,057,074	1,669	75,487	4x	16	cultivar	Clade 4 north	*in vitro*
S. tuberosum (Katahdin)	PDP_BR	1,116,684,092	1,671	75,137	4x	16	cultivar	Clade 4 north	*in vitro*
S. tuberosum (Kennebec)	PDP_BS	997,903,337	1,630	73,648	4x	16	cultivar	Clade 4 north	*in vitro*
S. tuberosum (MountainRose)	PDP_BT	977,228,032	1,831	72,834	4x	16	cultivar	Clade 4 north	*in vitro*
S. tuberosum (Norland)	PDP_BU	1,047,182,525	1,734	74,975	4x	16	cultivar	Clade 4 north	*in vitro*
S. tuberosum (PremierRusset)	PDP_BV	960,317,330	1,849	73,426	4x	16	cultivar	Clade 4 north	*in vitro*
S. tuberosum (PurpleMajesty)	PDP_BW	1,056,992,113	1,619	75,075	4x	16	cultivar	Clade 4 north	*in vitro*
S. tuberosum (RioGrandeRusset)	PDP_BX	983,629,693	1,886	73,853	4x	16	cultivar	Clade 4 north	*in vitro*
S. tuberosum (RussetBurbank)	PDP_BY	1,101,549,513	1,562	75,750	4x	16	cultivar	Clade 4 north	*in vitro*
S. tuberosum (Shepody)	PDP_BZ	979,562,054	1,874	73,739	4x	16	cultivar	Clade 4 north	*in vitro*
S. tuberosum (SierraGold)	PDP_CA	935,722,103	1,680	74,391	4x	16	cultivar	Clade 4 north	*in vitro*
S. tuberosum (Spunta)	PDP_CB	916,910,258	1,785	73,957	4x	16	cultivar	Clade 4 north	*in vitro*
S. tuberosum (YukonGold)	PDP_CC	930,096,131	1,929	74,017	4x	16	cultivar	Clade 4 north	*in vitro*
S. tuberosum Gp. *Andigena* (PI 214421)	PDP_CD	929,007,194	2,358	72,863	4x	16	landrace	Clade 4 north	Cultivated/landrace
S. phureja (CIP 703654)	PHU	873,028,468	6,471	65,226	2x	50	landrace	Clade 4 north	*in vitro*
S. phureja (PI 195191)	phu195191	733,166,000	2,979	60,429	2x	12	landrace	Clade 4 north	Cultivated/landrace
S. phureja (PI 195198)	phu195198	759,046,147	3,815	59,802	2x	12	landrace	Clade 4 north	Cultivated/landrace
S. phureja (PI 225665)	phu225665	761,128,960	2,780	61,273	2x	12	landrace	Clade 4 north	Cultivated/landrace
S. phureja (PI 225693)	phu225693	788,200,088	2,888	61,906	2x	12	landrace	Clade 4 north	Cultivated/landrace
S. phureja (PI 225703)	phu225703	805,751,047	3,101	61,368	2x	12	landrace	Clade 4 north	Cultivated/landrace

S. phureja (PI 243467)	phu243467	790,649,904	3,523	60,928	2x	12	landrace	Clade 4 north	Cultivated/landrace
S. phureja (PI 243468)	phu243468	779,498,620	2,729	62,557	2x	12	landrace	Clade 4 north	Cultivated/landrace
S. phureja (PI 243469)	phu243469	787,497,993	3,034	61,083	2x	12	landrace	Clade 4 north	Cultivated/landrace
S. phureja (PI 258855)	phu258855	748,495,294	2,801	62,235	2x	12	landrace	Clade 4 north	Cultivated/landrace
S. pinnatisectum (PI 253214)	pin253214	589,761,539	7,263	58,108	2x	12	wild	Clade 1+2	Clade 1+2
S. pinnatisectum (PI 537023)	pin537023	591,340,031	11,106	58,997	2x	12	wild	Clade 1+2	Clade 1+2
S. polyadenium (PI 161728)	pol161728	563,439,786	16,809	56,532	2x	12	wild	Clade 1+2	Clade 1+2
S. polyadenium (PI 347770)	pol347770	554,248,916	9,332	54,867	2x	12	wild	Clade 1+2	Clade 1+2
S. tuberosum (RH)	RH	NA	NA	74,362	2x	Hybrid	cultivar	Clade 4 north	*in vitro*
S. tuberosum (RussetNorkotah)	SCP_AA	1,179,661,935	1,874	NA	4x	16	cultivar	NA	NA
S. tuberosum (Missaukee)	SCP_AB	1,206,926,598	1,766	NA	4x	16	cultivar	NA	NA
S. tuberosum (Kalkaska)	SCP_AC	1,473,501,556	1,567	NA	4x	16	cultivar	NA	NA
S. tuberosum (Atlantic)	SCP_AD	1,319,594,511	1,695	NA	4x	16	cultivar	NA	NA
S. tuberosum (Snowden)	SCP_AE	1,244,274,578	1,809	NA	4x	16	cultivar	NA	NA
S. sogarandinum (PI 230510)	sog230510	718,416,512	4,507	64,999	2x	12	wild	NA	NA
S. sogarandinum (PI 365360)	sog365360	721,307,197	3,858	63,445	2x	12	wild	NA	NA
S. tuberosum (Solyntus)	Solyntus	716,171,447	63,701,590	66,506	4x	Hybrid	cultivar	Clade 4 north	*in vitro*
S. sparsipilum (PI 246536)	spa246536	732,266,494	5,142	65,115	2x	12	wild	Clade 4 south	South Wild
S. sparsipilum (PI 473375)	spa473375	763,692,119	3,865	64,835	2x	12	wild	Clade 4 south	South Wild
S. sparsipilum (PI 473385)	spa473385	759,488,633	3,548	64,524	2x	12	wild	Clade 4 south	South Wild
S. sparsipilum (PI 498134)	spa498134	751,158,744	2,949	63,650	2x	12	wild	Clade 4 south	South Wild
S. sparsipilum (PI 498284)	spa498284	741,261,929	2,595	63,052	2x	12	wild	Clade 4 south	South Wild
S. sparsipilum (PI 498285)	spa498285	764,560,390	2,875	65,313	2x	12	wild	Clade 4 south	South Wild
S. spegazzinii (PI 320299)	spe320299	731,745,746	2,766	63,180	2x	12	wild	Clade 4 south	South Wild
S. spegazzinii (PI 458335)	spe458335	767,966,551	3,741	64,649	2x	12	wild	Clade 4 south	South Wild
S. spegazzinii (PI 458337)	spe458337	717,617,431	3,904	63,553	2x	12	wild	Clade 4 south	South Wild
S. spegazzinii (PI 472966)	spe472966	751,736,661	4,088	65,085	2x	12	wild	Clade 4 south	South Wild
S. spegazzinii (PI 472988)	spe472988	766,836,524	2,343	63,191	2x	12	wild	Clade 4 south	South Wild
S. spegazzinii (PI 472990)	spe472990	750,705,546	3,620	64,960	2x	12	wild	Clade 4 south	South Wild
S. tuberosum (Spunta)	Spunta	2,219,201,069	1,081,689	75,831	4x	Hybrid	cultivar	Clade 4 north	*in vitro*
S. stenotomum (PI 195204)	ste195204	954,390,199	1,750	NA	2x	12	wild/landrace hybrid	NA	NA
S. stenotomum (PI 205527)	ste205527	779,290,382	2,728	63,220	2x	12	landrace	Clade 4 north	Cultivated/landrace
S. stenotomum (PI 230512)	ste230512	771,414,550	2,728	62,815	2x	12	landrace	Clade 4 north	Cultivated/landrace
S. stenotomum (PI 230513)	ste230513	780,199,427	2,957	61,861	2x	12	landrace	Clade 4 north	Cultivated/landrace
S. stenotomum (PI 234011)	ste234011	811,388,907	2,403	63,857	2x	12	landrace	Clade 4 north	Cultivated/landrace

S. stenophyllidium (PI 255527)	ste255527	742,645,881	4,190	56,496	2x	12	wild	Clade 1+2	Clade 1+2
S. stenotomum (PI 283141)	ste283141	836,327,228	2,649	64,300	2x	12	landrace	Clade 4 north	Cultivated/landrace
S. stenotomum (PI 365344)	ste365344	754,390,273	3,063	60,746	2x	12	landrace	Clade 4 north	Cultivated/landrace
S. stenotomum ssp. *stenotomum* (CIP 705834)	STN	944,999,932	5,861	67,738	2x	50	landrace	Clade 4 north	*in vitro*
S. verrucosum (PI 275260)	STP_DL_01	475,283,770	2,187	NA	2x	8	wild	NA	NA
S. tarijense (PI 217457)	tar217457	740,015,043	2,137	63,645	2x	12	wild	Clade 4 south	South Wild
S. tarijense (PI 414152)	tar414152	766,706,474	2,531	64,469	2x	12	wild	Clade 4 south	South Wild
S. tarijense (PI 458366)	tar458366	747,535,127	2,870	63,644	2x	12	wild	Clade 4 south	South Wild
S. tarijense (PI 473217)	tar473217	692,339,213	4,098	62,534	2x	12	wild	Clade 4 south	South Wild
S. tarijense (PI 473218)	tar473218	794,723,849	2,670	65,116	2x	12	wild	Clade 4 south	South Wild
S. tuberosum ssp. *tuberosum* (CIP 705053)	TBR	1,032,424,176	1,487	72,139	4x	50	landrace	Clade 4 north	*in vitro*
S. verrucosum (PI 195170)	ver195170	635,610,668	6,585	61,648	2x	12	wild	Clade 4 south	South Wild
S. verrucosum (PI 275256)	ver275256	635,278,015	7,458	62,663	2x	12	wild	Clade 4 south	South Wild
S. verrucosum (PI 275260)	ver275260	654,316,133	11,255	65,092	2x	12	wild	Clade 4 south	South Wild
S. vernei (PI 320332)	ver320332	730,957,924	4,363	64,031	2x	12	wild	Clade 4 south	South Wild
S. vernei (PI 320333)	ver320333	745,617,808	2,789	64,172	2x	12	wild	Clade 4 south	South Wild
S. vernei (PI 458370)	ver458370	762,481,748	4,500	65,677	2x	12	wild	Clade 4 south	South Wild
S. vernei (PI 473303)	ver473303	727,391,175	4,231	65,039	2x	12	wild	Clade 4 south	South Wild
S. vernei (PI 473309)	ver473309	726,423,546	5,221	66,401	2x	12	wild	Clade 4 south	South Wild
S. verrucosum (PI 498010)	ver498010	629,287,056	5,694	61,871	2x	12	wild	Clade 4 south	South Wild
S. verrucosum (PI 498061)	ver498061	640,341,066	5,823	62,112	2x	12	wild	Clade 4 south	South Wild
S. vernei (PI 500070)	ver500070	745,272,538	4,173	65,263	2x	12	wild	Clade 4 south	South Wild
S. verrucosum (PI 545745)	ver545745	648,112,201	5,129	62,163	2x	12	wild	Clade 4 south	South Wild
S. verrucosum (PI 545747)	ver545747	649,815,967	7,565	63,574	2x	12	wild	Clade 4 south	South Wild
S. microdontum (PI 545884)	ver545884	899,101,532	2,108	67,138	2x	12	wild	Clade 4 south	South Wild
S. vernei (PI 558150)	ver558150	730,698,831	3,751	64,403	2x	12	wild	Clade 4 south	South Wild
S. verrucosum (PI 558463)	ver558463	649,281,279	8,620	63,524	2x	12	wild	Clade 4 south	South Wild
S. verrucosum (PI 558488)	ver558488	646,947,343	6,961	62,819	2x	12	wild	Clade 4 south	South Wild
S. violaceimarmoratum (PI 473396)	vio473396	714,039,139	3,788	63,352	2x	12	wild	Clade 4 north	North Wild
S. violaceimarmoratum (PI 473398)	vio473398	766,800,527	3,249	63,485	2x	12	wild	Clade 4 north	North Wild
S. violaceimarmoratum (PI 498296)	vio498296	699,872,821	2,396	61,316	2x	12	wild	Clade 4 north	North Wild

S. phureja x *S. tuberosum* (W5281.2)	W5281.2	872,319,509	40,318	65,974	2x	50	cultivar	Clade 4 north	*in vitro*

*Hybrid - Combination of long and short reads used in constructing the assembly

Table S2. A list of accessions used in this study with their raw read and assembly IDs used for building the pan-genome.

Genome	Assembly available	Assembly	SRA id	Biosample	Publication
S. tuberosum (07506-01)	Yes	JAKMHP000000000	SRR14482391	SAMN17059157	(1)
S. tuberosum (08675-21)	Yes	JAKMHQ000000000	SRR14482390	SAMN17059158	(1)
S. tuberosum (10908-06)	Yes	JAKMHS000000000	SRR14482388	SAMN17059160	(1)
S. tuberosum (11379-03)	Yes	JAKMHR000000000	SRR14482389	SAMN17059159	(1)
S. tuberosum (12120-03)	Yes	JAKMHV000000000	SRR14482385	SAMN17059163	(1)
S. andigena (12625-02)	Yes	JAKMHU000000000	SRR14482386	SAMN17059162	(1)
S. abancayense (PI 458403)	No	-	SRR7716081	SAMN07540429	(2)
S. abancayense (PI 458404)	No	-	SRR7716082	SAMN07540430	(2)
S. achacachense (PI 558032)	No	-	SRR7716083	SAMN07540512	(2)
S. acroglossum (PI 365313)	No	-	SRR5959448	SAMN07540368	(2)
S. acroscopicum (PI 365314)	No	-	SRR5959449	SAMN07540369	(2)
S. acroglossum (PI 498204)	No	-	SRR5959457	SAMN07540377	(2)
S. tuberosum ssp. *andigena* (CIP 700921)	Yes	WBIE00000000	SRR10248515	SAMN12684893	(3)
S. tuberosum ssp. *andigena* (CIP 702853)	Yes	WBIF00000000	SRR10248514	SAMN12684894	(3)
S. ajanhuiri (CIP 703810)	Yes	WBIA00000000	SRR10244437	SAMN12684889	(4)
S. albornozii (PI 498206)	No	-	SRR5959458	SAMN07540378	(2)
S. tuberosum (Altus)	Yes	https://doi.org/10.5061/dryad.3n5tb2rhw	SRR14993628, SRR14993639, SRR14993640	SAMN19812628	(5)
S. ambosinum (PI 365317)	No	-	SRR7716084	SAMN07540414	(2)
S. ambosinum (PI 365362)	No	-	SRR7716085	SAMN07540417	(2)
S. ambosinum (PI 498209)	No	-	SRR7716086	SAMN07540480	(2)
S. ambosinum (PI 498210)	No	-	SRR7716087	SAMN07540481	(2)
S. ambosinum (PI 498212)	No	-	SRR7716088	SAMN07540482	(2)
S. ambosinum (PI 498213)	No	-	SRR7716089	SAMN07540483	(2)
S. andreanum (PI 320345)	No	-	SRR5959447	SAMN07540367	(2)
S. andreanum (PI 561648)	No	-	SRR5959462	SAMN07540382	(2)
S. andreanum (PI 561658)	No	-	SRR7716090	SAMN07540383	(2)
S. tuberosum (Atlantic)	Yes	https://doi.org/10.5061/dryad.3n5tb2rhw	SRR14807269, SRR14807268, SRR14807262	SAMN19655101	(5)

S. tuberosum (Avenger)	Yes	https://doi.org/10.5061/dryad.3n5tb2rhw	SRR14993622, SRR14993623	SAMN19812629	(5)
S. avilesii (PI 498091)	No	-	SRR7716091	SAMN07540474	(2)
S. avilesii (PI 498092)	No	-	SRR7716092	SAMN07540475	(2)
S. avilesii (PI 498093)	No	-	SRR7716093	SAMN07540476	(2)
S. microdontum (PI 218225)	No	-	SRR7716094	SAMN07540393	(2)
S. berthaultii (PI 498105)	No	-	SRR7716095	SAMN07540477	(2)
S. berthaultii (PI 527886)	No	-	SRR7716096	SAMN07540494	(2)
S. berthaultii (PI 545850)	No	-	SRR7716097	SAMN07540498	(2)
S. blanco (PI 498214)	No	-	SRR5959459	SAMN07540379	(2)
S. stenophyllidium (PI 320265)	No	-	SRR5957604	SAMN07540356	(2)
S. stenophyllidium (PI 558460)	No	-	SRR5958650	SAMN07540360	(2)
S. brevicaule (PI 310931)	No	-	SRR7716098	SAMN07540407	(2)
S. brevicaule (PI 473378)	No	-	SRR7716099	SAMN07540461	(2)
S. brevicaule (PI 498111)	No	-	SRR7716100	SAMN07540478	(2)
S. brevicaule (PI 498218)	No	-	SRR7716101	SAMN07540484	(2)
S. brevicaule (PI 545968)	No	-	SRR7716102	SAMN07540502	(2)
S. brevicaule (PI 545970)	No	-	SRR7716103	SAMN07540503	(2)
S. brevicaule (PI 545971)	No	-	SRR7716104	SAMN07540504	(2)
S. brevicaule (PI 545981)	No	-	SRR7716105	SAMN07540508	(2)
S. bukasovii (CIP 761748)	Yes	WBIB00000000	SRR10244436	SAMN12684890	(4)
S. bukasovii (CIP 761748)	Yes	-	SRR19432688	SAMN13916279	(6)
S. bukasovii (PI 266385)	No	-	SRR7716106	SAMN07540400	(2)
S. bukasovii (PI 365353)	No	-	SRR7716107	SAMN07540415	(2)
S. bukasovii (PI 414155)	No	-	SRR7716110	SAMN07540419	(2)
S. bukasovii (PI 473492)	No	-	SRR7716111	SAMN07540466	(2)
S. bukasovii (PI 473493)	No	-	SRR7716112	SAMN07540467	(2)
S. bukasovii (PI 473494)	No	-	SRR7716113	SAMN07540468	(2)
S. bukasovii (PI 568933)	No	-	SRR7716114	SAMN07540519	(2)
S. bukasovii (PI 568954)	No	-	SRR7716115	SAMN07540520	(2)
S. bulbocastanum (PI 545751)	No	-	SRR5958649	SAMN07540359	(2)
S. bulbocastanum (PI 604074)	No	-	SRR5958651	SAMN07540361	(2)
S. cajamarquense (PI 230522)	No	-	SRR5959444	SAMN07540364	(2)

S. canasense (PI 210035)	No	-	SRR7716116	SAMN07540386	(2)
S. canasense (PI 246533)	No	-	SRR7716117	SAMN07540396	(2)
S. canasense (PI 265864)	No	-	SRR7716118	SAMN07540398	(2)
S. canasense (PI 265865)	No	-	SRR7716119	SAMN07540399	(2)
S. canasense (PI 283084)	No	-	SRR7716120	SAMN07540406	(2)
S. canasense (PI 442696)	No	-	SRR7716121	SAMN07540420	(2)
S. canasense (PI 473355)	No	-	SRR7716122	SAMN07540458	(2)
S. canasense (PI 498226)	No	-	SRR7716123	SAMN07540485	(2)
S. canasense (PI 498227)	No	-	SRR7716124	SAMN07540486	(2)
S. canasense (PI 545972)	No	-	SRR7716125	SAMN07540505	(2)
S. canasense (PI 568969)	No	-	SRR7716126	SAMN07540552	(2)
S. cardiophyllum (PI 283062)	No	-	SRR7716127	SAMN07540544	(2)
S. cardiophyllum (PI 283063)	No	-	SRR7716128	SAMN07540545	(2)
S. cardiophyllum (PI 347759)	No	-	SRR7716129	SAMN07540547	(2)
S. tuberosum (CastleRusset)	Yes	https://doi.org/10.5061/dryad.3n5tb2rhw	SRR14807263, SRR14807264	SAMN19655102	(5)
S. chaucha (CIP 707129)	Yes	WBIC00000000	SRR10248511	SAMN12684891	(3)
S. chacoense (PI 275138)	No	-	SRR7716130	SAMN07540401	(2)
S. chacoense (PI 320294)	No	-	SRR7716131	SAMN07540410	(2)
S. chacoense (PI 472816)	No	-	SRR7716132	SAMN07540431	(2)
S. chacoense (PI 472830)	No	-	SRR7716133	SAMN07540432	(2)
S. chacoense (PI 500020)	No	-	SRR7716134	SAMN07540489	(2)
S. chomatophilum (PI 365328)	No	-	SRR5959450	SAMN07540370	(2)
S. chomatophilum (PI 365339)	No	-	SRR5959454	SAMN07540374	(2)
S. tuberosum (Colomba)	Yes	https://doi.org/10.5061/dryad.3n5tb2rhw	SRR14993638, SRR14993610, SRR14993609	SAMN19812630	(5)
S. commersonii (COM)	Yes	JXZD01000000	SRR12407221, SRR12407222	SAMN15755581	(7)
S. curtilobum (CIP 702937)	Yes	WBIH00000000	SRR10248510	SAMN12684896	(3)
S. tuberosum Gp. *Phureja* (DM)	Yes	http://spuddb.uga.edu/dm_v6_1_download.shtml	SRR11908546	SAMN15077101	(8)
S. tuberosum (DW84-1457)	Yes	JAKMHT000000000	SRR14482387	SAMN17059161	(1)
S. stenotomum ssp. *goniocalyx* (CIP 702472)	Yes	WBHW00000000	SRR10244441	SAMN12345900	(4)

S. goniocalyx (PI 195186)	No	-	SRR7716136	SAMN07540521	(2)
S. goniocalyx (PI 195188)	No	-	SRR7716137	SAMN07540522	(2)
S. goniocalyx (PI 195214)	No	-	SRR7716138	SAMN07540526	(2)
S. stenotomum ssp. *goniocalyx* (CIP 704393)	Yes	WBHX00000000	SRR10244440	SAMN12684886	(4)
S. goniocalyx (PI 458393)	No	-	SRR7716139	SAMN07540541	(2)
S. gourlayi (PI 472911)	No	-	SRR7716140	SAMN07540433	(2)
S. gourlayi (PI 472991)	No	-	SRR7716141	SAMN07540441	(2)
S. gourlayi (PI 472995)	No	-	SRR7716142	SAMN07540442	(2)
S. gourlayi (PI 473019)	No	-	SRR7716143	SAMN07540443	(2)
S. gourlayi (PI 473077)	No	-	SRR7716144	SAMN07540448	(2)
S. gourlayi (PI 473106)	No	-	SRR7716145	SAMN07540449	(2)
S. gourlayi (PI 500022)	No	-	SRR7716146	SAMN07540490	(2)
S. gourlayi (PI 537026)	No	-	SRR7716147	SAMN07540495	(2)
S. gourlayi (PI 545865)	No	-	SRR7716148	SAMN07540499	(2)
S. gourlayi (PI 545975)	No	-	SRR7716149	SAMN07540506	(2)
S. gourlayi (PI 545978)	No	-	SRR7716150	SAMN07540507	(2)
S. gourlayi (PI 558067)	No	-	SRR7716151	SAMN07540513	(2)
S. tuberosum (H412-1)	Yes	JAKMHO000000000	SRR14482392	SAMN17059155	(1)
S. stenotomum (PI 320364)	No	-	SRR7716152	SAMN07540539	(2)
S. hondelmannii (PI 473365)	No	-	SRR7716153	SAMN07540459	(2)
S. hondelmannii (PI 498067)	No	-	SRR7716154	SAMN07540472	(2)
S. hondelmannii (PI 498071)	No	-	SRR7716155	SAMN07540473	(2)
S. hondelmannii (PI 545879)	No	-	SRR7716156	SAMN07540500	(2)
S. kurtzianum (PI 320327)	No	-	SRR5959446	SAMN07540366	(2)
S. marinasense (PI 498255)	No	-	SRR5959460	SAMN07540380	(2)
S. hypacrarthrum (PI 473477)	No	-	SRR5959455	SAMN07540375	(2)
S. incamayoense (PI 473060)	No	-	SRR7716157	SAMN07540444	(2)
S. incamayoense (PI 473067)	No	-	SRR7716158	SAMN07540445	(2)
S. incamayoense (PI 473069)	No	-	SRR7716159	SAMN07540446	(2)
S. incamayoense (PI 473070)	No	-	SRR7716160	SAMN07540447	(2)
S. incamayoense (PI 500048)	No	-	SRR7716161	SAMN07540492	(2)
S. jamesii (PI 641944)	No	-	SRR5958652	SAMN07540362	(2)
S. jamesii (PI 664024)	No	-	SRR5959443	SAMN07540363	(2)

S. juzepczukii (CIP 706050)	Yes	WBID00000000	SRR10248512	SAMN12684892	(3)
S. kurtzianum (PI 472924)	No	-	SRR7716162	SAMN07540434	(2)
S. kurtzianum (PI 472936)	No	-	SRR7716163	SAMN07540435	(2)
S. kurtzianum (PI 472948)	No	-	SRR7716164	SAMN07540436	(2)
S. kurtzianum (PI 472952)	No	-	SRR7716165	SAMN07540437	(2)
S. kurtzianum (PI 558185)	No	-	SRR7716166	SAMN07540515	(2)
S. kurtzianum (PI 558208)	No	-	SRR7716167	SAMN07540516	(2)
S. laxissimum (PI 283088)	No	-	SRR7716168	SAMN07540546	(2)
S. laxissimum (PI 498252)	No	-	SRR7716169	SAMN07540550	(2)
S. laxissimum (PI 607887)	No	-	SRR7716170	SAMN07540553	(2)
S. leptophyes (PI 458378)	No	-	SRR7716171	SAMN07540426	(2)
S. leptophyes (PI 473342)	No	-	SRR7716172	SAMN07540455	(2)
S. leptophyes (PI 473446)	No	-	SRR7716173	SAMN07540463	(2)
S. leptophyes (PI 473451)	No	-	SRR7716174	SAMN07540464	(2)
S. leptophyes (PI 545985)	No	-	SRR7716175	SAMN07540509	(2)
S. leptophyes (PI 545987)	No	-	SRR7716176	SAMN07540510	(2)
S. limbaniense (PI 473468)	No	-	SRR7716177	SAMN07540465	(2)
S. chacoense (M6)	Yes	https://datadryad.org/stash/dataset/doi:10.5061/dryad.kc835	SRR5264013,SRR5264014,SRR5264018,SRR5264019,SRR5264020	SAMN06338130	(9)
S. marinasense (PI 210040)	No	-	SRR7716178	SAMN07540387	(2)
S. marinasense (PI 310944)	No	-	SRR7716179	SAMN07540408	(2)
S. medians (PI 210045)	No	-	SRR7716180	SAMN07540389	(2)
S. medians (PI 230507)	No	-	SRR7716181	SAMN07540394	(2)
S. medians (PI 320260)	No	-	SRR7716182	SAMN07540409	(2)
S. medians (PI 458402)	No	-	SRR7716183	SAMN07540428	(2)
S. medians (PI 473496)	No	-	SRR7716184	SAMN07540469	(2)
S. megistacrolobum (PI 210034)	No	-	SRR7716185	SAMN07540385	(2)
S. megistacrolobum (PI 473158)	No	-	SRR7716186	SAMN07540450	(2)
S. megistacrolobum (PI 500029)	No	-	SRR7716187	SAMN07540491	(2)
S. megistacrolobum (PI 546000)	No	-	SRR7716188	SAMN07540511	(2)
S. multiinterruptum (PI 210044)	No	-	SRR7716189	SAMN07540388	(2)

S. multidissectum (PI 210052)	No	-	SRR7716190	SAMN07540390	(2)
S. multidissectum (PI 210055)	No	-	SRR7716191	SAMN07540391	(2)
S. multidissectum (PI 275272)	No	-	SRR5959445	SAMN07540365	(2)
S. multiinterruptum (PI 365336)	No	-	SRR5959451	SAMN07540371	(2)
S. multiinterruptum (PI 365337)	No	-	SRR5959452	SAMN07540372	(2)
S. multiinterruptum (PI 365338)	No	-	SRR5959453	SAMN07540373	(2)
S. multidissectum (PI 473349)	No	-	SRR7716192	SAMN07540456	(2)
S. multidissectum (PI 473352)	No	-	SRR7716193	SAMN07540457	(2)
S. multiinterruptum (PI 498266)	No	-	SRR5959461	SAMN07540381	(2)
S. tuberosum (Superior)	No	-	SRR5349638	SAMN06564622	(10)
S. okadae (OKA15)	Yes	JAKMHW000000000	SRR14482384	SAMN17860560	(1)
S. pampasense (PI 275274)	No	-	SRR7716195	SAMN07540404	(2)
S. pampasense (PI 275275)	No	-	SRR7716196	SAMN07540405	(2)
S. pampasense (PI 442697)	No	-	SRR7716197	SAMN07540421	(2)
S. pampasense (PI 458381)	No	-	SRR7716198	SAMN07540427	(2)
S. paucissectum (PI 473489)	No	-	SRR5959456	SAMN07540376	(2)
S. phureja (PI 195191)	No	-	SRR5349637	SAMN06564684	(10)
S. stenotomum (PI 195204)	No	-	SRR5349636	SAMN06564685	(10)
S. multidissectum (PI 210044)	No	-	SRR5349635	SAMN06564686	(10)
S. phureja (PI 225710)	No	-	SRR5349634	SAMN06564687	(10)
S. stenotomum (PI 230512)	No	-	SRR5349633	SAMN06564688	(10)
S. stenotomum (PI 234011)	No	-	SRR5349632	SAMN06564689	(10)
S. stenotomum (PI 234013)	No	-	SRR5349631	SAMN06564690	(10)
S. phureja (PI 243469)	No	-	SRR5349630	SAMN06564691	(10)
S. bukasovii (PI 265863)	No	-	SRR5349629	SAMN06564692	(10)
S. medians (PI 265872)	No	-	SRR5349628	SAMN06564693	(10)
S. chacoense (PI 275139)	No	-	SRR5349627	SAMN06564694	(10)
S. stenotomum (PI 292110)	No	-	SRR5349626	SAMN06564695	(10)
S. raphanifolium (PI 296126)	No	-	SRR5349625	SAMN06564696	(10)
S. phureja (PI 320355)	No	-	SRR5349624	SAMN06564697	(10)
S. stenotomum (PI 365344)	No	-	SRR5349623	SAMN06564698	(10)
S. infundibuliforme (PI 458324)	No	-	SRR5349622	SAMN06564699	(10)
S. microdontum (PI 458355)	No	-	SRR5349621	SAMN06564700	(10)

S. berthaultii (PI 458365)	No	-	SRR5349620	SAMN06564701	(10)
S. okadae (PI 458368)	No	-	SRR5349619	SAMN06564702	(10)
S. spegazzinii (PI 472978)	No	-	SRR5349618	SAMN06564703	(10)
S. gourlayi (PI 473065)	No	-	SRR5349617	SAMN06564704	(10)
S. vernei (PI 473305)	No	-	SRR5349616	SAMN06564705	(10)
S. sparsipilum (PI 473385)	No	-	SRR5349615	SAMN06564706	(10)
S. brevicaule (PI 498112)	No	-	SRR5349614	SAMN06564707	(10)
S. kurtzianum (PI 498359)	No	-	SRR5349613	SAMN06564708	(10)
S. boliviense (PI 545964)	No	-	SRR5349612	SAMN06564709	(10)
S. leptophyes (PI 545987)	No	-	SRR5349611	SAMN06564710	(10)
S. megistracrolobum (PI 546000)	No	-	SRR5349610	SAMN06564711	(10)
S. commersonii (PI 558050)	No	-	SRR5349609	SAMN06564712	(10)
S. tuberosum Gp. *Chilotanum* (PI 245847)	No	-	SRR5349608	SAMN06564713	(10)
S. tuberosum Gp. *Andigena* (PI 245935)	No	-	SRR5349607	SAMN06564714	(10)
S. tuberosum Gp. *Andigena* (PI 245940)	No	-	SRR5349606	SAMN06564715	(10)
S. tuberosum Gp. *Andigena* (PI 258874)	No	-	SRR5349605	SAMN06564716	(10)
S. tuberosum Gp. *Chilotanum* (PI 258885)	No	-	SRR5349604	SAMN06564717	(10)
S. tuberosum Gp. *Andigena* (PI 365345)	No	-	SRR5349603	SAMN06564718	(10)
S. tuberosum Gp. *Andigena* (PI 546023)	No	-	SRR5349602	SAMN06564719	(10)
S. tuberosum Gp. *Andigena* (PI 558142)	No	-	SRR5349601	SAMN06564720	(10)
S. tuberosum Gp. *Andigena* (PI 607886)	No	-	SRR5349600	SAMN06564721	(10)
S. tuberosum (Burbank)	No	-	SRR5349599	SAMN06564722	(10)
S. tuberosum (DakotaDiamond)	No	-	SRR5349598	SAMN06564723	(10)
S. tuberosum (EarlyRose)	No	-	SRR5349597	SAMN06564724	(10)
S. tuberosum (GarnetChili)	No	-	SRR5349596	SAMN06564725	(10)
S. tuberosum (IrishCobbler)	No	-	SRR5349595	SAMN06564726	(10)
S. tuberosum (Katahdin)	No	-	SRR5349594	SAMN06564727	(10)
S. tuberosum (Kennebec)	No	-	SRR5349593	SAMN06564728	(10)
S. tuberosum (MountainRose)	No	-	SRR5349592	SAMN06564729	(10)
S. tuberosum (Norland)	No	-	SRR5349591	SAMN06564730	(10)
S. tuberosum (PremierRusset)	No	-	SRR5349590	SAMN06564731	(10)
S. tuberosum (PurpleMajesty)	No	-	SRR5349589	SAMN06564732	(10)
S. tuberosum (RioGrandeRusset)	No	-	SRR5349588	SAMN06564733	(10)

S. tuberosum (RussetBurbank)	No	-	SRR5349587	SAMN06564734	(10)
S. tuberosum (Shepody)	No	-	SRR5349586	SAMN06564735	(10)
S. tuberosum (SierraGold)	No	-	SRR5349585	SAMN06564736	(10)
S. tuberosum (Spunta)	No	-	SRR5349584	SAMN06564737	(10)
S. tuberosum (YukonGold)	No	-	SRR5349583	SAMN06564738	(10)
S. tuberosum Gp. *Andigena* (PI 214421)	No	-	SRR5349582	SAMN06564739	(10)
S. phureja (CIP 703654)	Yes	WBHY00000000	SRR10244439	SAMN12684887	(4)
S. phureja (PI 195191)	No	-	SRR7716199	SAMN07540523	(2)
S. phureja (PI 195198)	No	-	SRR7716200	SAMN07540524	(2)
S. phureja (PI 225665)	No	-	SRR7716201	SAMN07540528	(2)
S. phureja (PI 225693)	No	-	SRR7716202	SAMN07540529	(2)
S. phureja (PI 225703)	No	-	SRR7716203	SAMN07540530	(2)
S. phureja (PI 243467)	No	-	SRR7716204	SAMN07540534	(2)
S. phureja (PI 243468)	No	-	SRR7716205	SAMN07540535	(2)
S. phureja (PI 243469)	No	-	SRR7716206	SAMN07540536	(2)
S. phureja (PI 258855)	No	-	SRR7716207	SAMN07540537	(2)
S. pinnatisectum (PI 253214)	No	-	SRR5957602	SAMN07540354	(2)
S. pinnatisectum (PI 537023)	No	-	SRR5958648	SAMN07540358	(2)
S. polyadenium (PI 161728)	No	-	SRR5957601	SAMN07367888	(2)
S. polyadenium (PI 347770)	No	-	SRR5958639	SAMN07540357	(2)
S. tuberosum (RH)	Yes	http://spuddb.uga.edu/rh_potato_download.shtml	SRR10376155, SRR10376166, SRR10376177, SRR10376178	SAMN12826500	(11)
S. tuberosum (RussetNorkotah)	No	-	SRR5349581	SAMN06564740	(10)
S. tuberosum (Missaukee)	No	-	SRR5349580	SAMN06564741	(10)
S. tuberosum (Kalkaska)	No	-	SRR5349579	SAMN06564742	(10)
S. tuberosum (Atlantic)	No	-	SRR5349578	SAMN06564623	(10)
S. tuberosum (Snowden)	No	-	SRR5349577	SAMN06564743	(10)
S. sogarandinum (PI 230510)	No	-	SRR7716208	SAMN07540395	(2)
S. sogarandinum (PI 365360)	No	-	SRR7716209	SAMN07540416	(2)
S. tuberosum (Solyntus)	Yes	https://www.plantbreeding.wur.nl/Solyntus/#download	SRR12191833	SAMN15221885	(12)
S. sparsipilum (PI 246536)	No	-	SRR7716210	SAMN07540397	(2)

S. sparsipilum (PI 473375)	No	-	SRR7716211	SAMN07540460	(2)
S. sparsipilum (PI 473385)	No	-	SRR7716212	SAMN07540462	(2)
S. sparsipilum (PI 498134)	No	-	SRR7716213	SAMN07540479	(2)
S. sparsipilum (PI 498284)	No	-	SRR7716214	SAMN07540487	(2)
S. sparsipilum (PI 498285)	No	-	SRR7716215	SAMN07540488	(2)
S. spegazzinii (PI 320299)	No	-	SRR7716216	SAMN07540411	(2)
S. spegazzinii (PI 458335)	No	-	SRR7716217	SAMN07540422	(2)
S. spegazzinii (PI 458337)	No	-	SRR7716218	SAMN07540423	(2)
S. spegazzinii (PI 472966)	No	-	SRR7716219	SAMN07540438	(2)
S. spegazzinii (PI 472988)	No	-	SRR7716220	SAMN07540439	(2)
S. spegazzinii (PI 472990)	No	-	SRR7716221	SAMN07540440	(2)
S. tuberosum (Spunta)	Yes	https://doi.org/10.5061/dryad.3n5tb2rhw	SRR14993631, SRR14993632	SAMN19812631	(5)
S. stenotomum (PI 195204)	No	-	SRR7716222	SAMN07540525	(2)
S. stenotomum (PI 205527)	No	-	SRR7716223	SAMN07540527	(2)
S. stenotomum (PI 230512)	No	-	SRR7716224	SAMN07540531	(2)
S. stenotomum (PI 230513)	No	-	SRR7716225	SAMN07540532	(2)
S. stenotomum (PI 234011)	No	-	SRR7716226	SAMN07540533	(2)
S. stenophyllidium (PI 255527)	No	-	SRR5957603	SAMN07540355	(2)
S. stenotomum (PI 283141)	No	-	SRR7716227	SAMN07540538	(2)
S. stenotomum (PI 365344)	No	-	SRR7716228	SAMN07540540	(2)
S. stenotomum ssp. *stenotomum* (CIP 705834)	Yes	WBHZ00000000	SRR10244438	SAMN12684888	(4)
S. verrucosum (PI 275260)	No	-	SRR5349572	SAMN06564748	(10)
S. tarijense (PI 217457)	No	-	SRR7716229	SAMN07540392	(2)
S. tarijense (PI 414152)	No	-	SRR7716230	SAMN07540418	(2)
S. tarijense (PI 458366)	No	-	SRR7716231	SAMN07540424	(2)
S. tarijense (PI 473217)	No	-	SRR7716232	SAMN07540451	(2)
S. tarijense (PI 473218)	No	-	SRR7716233	SAMN07540452	(2)
S. tuberosum ssp. *tuberosum* (CIP 705053)	Yes	WBIG000000000	SRR10248513	SAMN12684895	(3)
S. verrucosum (PI 195170)	No	-	SRR7716299	SAMN07540384	(2)
S. verrucosum (PI 275256)	No	-	SRR7716300	SAMN07540402	(2)
S. verrucosum (PI 275260)	No	-	SRR7716301	SAMN07540403	(2)
S. vernei (PI 320332)	No	-	SRR7716302	SAMN07540412	(2)

S. vernei (PI 320333)	No	-	SRR7716303	SAMN07540413	(2)
S. vernei (PI 458370)	No	-	SRR7716304	SAMN07540425	(2)
S. vernei (PI 473303)	No	-	SRR7716305	SAMN07540453	(2)
S. vernei (PI 473309)	No	-	SRR7716306	SAMN07540454	(2)
S. verrucosum (PI 498010)	No	-	SRR7716307	SAMN07540470	(2)
S. verrucosum (PI 498061)	No	-	SRR7716308	SAMN07540471	(2)
S. vernei (PI 500070)	No	-	SRR7716309	SAMN07540493	(2)
S. verrucosum (PI 545745)	No	-	SRR7716310	SAMN07540496	(2)
S. verrucosum (PI 545747)	No	-	SRR7716311	SAMN07540497	(2)
S. microdontum (PI 545884)	No	-	SRR7716312	SAMN07540501	(2)
S. vernei (PI 558150)	No	-	SRR7716313	SAMN07540514	(2)
S. verrucosum (PI 558463)	No	-	SRR7716314	SAMN07540517	(2)
S. verrucosum (PI 558488)	No	-	SRR7716315	SAMN07540518	(2)
S. violaceimarmoratum (PI 473396)	No	-	SRR7716316	SAMN07540548	(2)
S. violaceimarmoratum (PI 473398)	No	-	SRR7716317	SAMN07540549	(2)
S. violaceimarmoratum (PI 498296)	No	-	SRR7716318	SAMN07540551	(2)
S. phureja x *S. tuberosum* (W5281.2)	Yes	JAKMHN000000000	SRR14482393	SAMN17059154	(1)

Table S3. List of accession numbers of the RNA-seq data retrieved from NCBI (on Aug 4th 2021).

SRA ID	BioProject ID	Reference
SRR14134355	PRJNA713962	https://www.ncbi.nlm.nih.gov/bioproject/PRJNA713962
SRR14134353	PRJNA713962	https://www.ncbi.nlm.nih.gov/bioproject/PRJNA713962
SRR14134351	PRJNA713962	https://www.ncbi.nlm.nih.gov/bioproject/PRJNA713962
SRR14134365	PRJNA713962	https://www.ncbi.nlm.nih.gov/bioproject/PRJNA713962
SRR14134363	PRJNA713962	https://www.ncbi.nlm.nih.gov/bioproject/PRJNA713962
SRR14134361	PRJNA713962	https://www.ncbi.nlm.nih.gov/bioproject/PRJNA713962
SRR14134359	PRJNA713962	https://www.ncbi.nlm.nih.gov/bioproject/PRJNA713962
SRR14134357	PRJNA713962	https://www.ncbi.nlm.nih.gov/bioproject/PRJNA713962
SRR12284159	PRJNA647649	(13)
SRR12284156	PRJNA647649	(13)
SRR12104879	PRJNA641265	(14)
SRR12104876	PRJNA641265	(14)
SRR12104873	PRJNA641265	(14)
SRR12104842	PRJNA641265	(14)
SRR12104856	PRJNA641265	(14)
SRR12104853	PRJNA641265	(14)
SRR12104850	PRJNA641265	(14)
SRR12104846	PRJNA641265	(14)
SRR12104863	PRJNA641265	(14)
SRR10549529	PRJNA591474	(15)
SRR10549512	PRJNA591474	(15)
SRR10599080	PRJNA592879	https://www.ncbi.nlm.nih.gov/bioproject/PRJNA592879
SRR10599093	PRJNA592879	https://www.ncbi.nlm.nih.gov/bioproject/PRJNA592879
SRR10599090	PRJNA592879	https://www.ncbi.nlm.nih.gov/bioproject/PRJNA592879
SRR10599097	PRJNA592879	https://www.ncbi.nlm.nih.gov/bioproject/PRJNA592879
SRR10599086	PRJNA592879	https://www.ncbi.nlm.nih.gov/bioproject/PRJNA592879
SRR10599083	PRJNA592879	https://www.ncbi.nlm.nih.gov/bioproject/PRJNA592879
SRR10436903	PRJNA589113	(16)
SRR10376106	PRJNA573826	(11)
SRR10376109	PRJNA573826	(11)
SRR10376113	PRJNA573826	(11)
SRR10376116	PRJNA573826	(11)
SRR10376119	PRJNA573826	(11)
SRR10376132	PRJNA573826	(11)
SRR10376136	PRJNA573826	(11)
SRR9204643	PRJNA546591	(17)
SRR9204646	PRJNA546591	(17)

SRR9204645	PRJNA546591	(17)
SRR8892943	PRJNA532699	(18)
SRR8892942	PRJNA532699	(18)
SRR8389894	PRJNA512866	https://www.ncbi.nlm.nih.gov/bioproject/PRJNA512866
SRR8389892	PRJNA512866	https://www.ncbi.nlm.nih.gov/bioproject/PRJNA512866
SRR8389898	PRJNA512866	https://www.ncbi.nlm.nih.gov/bioproject/PRJNA512866
SRR8389896	PRJNA512866	https://www.ncbi.nlm.nih.gov/bioproject/PRJNA512866
SRR7651448	PRJNA484851	https://www.ncbi.nlm.nih.gov/bioproject/PRJNA484851
SRR7651449	PRJNA484851	https://www.ncbi.nlm.nih.gov/bioproject/PRJNA484851
SRR5970137	PRJNA386512	(19)
SRR5970131	PRJNA386512	(19)
SRR5970145	PRJNA386512	(19)
SRR5970135	PRJNA386512	(19)
SRR5970133	PRJNA386512	(19)
SRR5970129	PRJNA386512	(19)
SRR5970143	PRJNA386512	(19)
SRR5970139	PRJNA386512	(19)
SRR5970141	PRJNA386512	(19)
SRR5970149	PRJNA386512	(19)
SRR5970147	PRJNA386512	(19)
SRR5970151	PRJNA386512	(19)
SRR5195768	PRJNA362370	(9)
SRR5195766	PRJNA362370	(9)
SRR5195765	PRJNA362370	(9)
SRR3656926	PRJNA325087	https://www.ncbi.nlm.nih.gov/bioproject/PRJNA325087
SRR3656918	PRJNA325087	https://www.ncbi.nlm.nih.gov/bioproject/PRJNA325087
SRR2782412	PRJNA299204	(20)
SRR2782581	PRJNA299204	(20)
SRR2782690	PRJNA299204	(20)
SRR2782413	PRJNA299204	(20)
SRR2782438	PRJNA299204	(20)
SRR2782414	PRJNA299204	(20)
SRR2782417	PRJNA299204	(20)
SRR1233155	PRJNA244102	https://www.ncbi.nlm.nih.gov/bioproject/PRJNA244102
SRR1233157	PRJNA244102	https://www.ncbi.nlm.nih.gov/bioproject/PRJNA244102
SRR1233154	PRJNA244102	https://www.ncbi.nlm.nih.gov/bioproject/PRJNA244102
SRR1233156	PRJNA244102	https://www.ncbi.nlm.nih.gov/bioproject/PRJNA244102
SRR1687231	PRJNA269013	(7)
SRR1687232	PRJNA269013	(7)

SRR1039535	PRJNA229434	(21)
SRR1103933	PRJNA229434	(21)
SRR1103934	PRJNA229434	(21)
SRR1232054	PRJNA244321	https://www.ncbi.nlm.nih.gov/bioproject/PRJNA244321
SRR1233162	PRJNA244102	https://www.ncbi.nlm.nih.gov/bioproject/PRJNA244102
SRR1233166	PRJNA244102	https://www.ncbi.nlm.nih.gov/bioproject/PRJNA244102
SRR1687229	PRJNA269013	(7)
SRR1687230	PRJNA269013	(7)
SRR122133	PRJNA63145	(22)
SRR122129	PRJNA63145	(22)
SRR122113	PRJNA63145	(22)
SRR122109	PRJNA63145	(22)
SRR122122	PRJNA63145	(22)
SRR122124	PRJNA63145	(22)
SRR122139	PRJNA63145	(22)
SRR10153124	PRJNA573056	https://www.ncbi.nlm.nih.gov/bioproject/PRJNA573056
SRR10153125	PRJNA573056	https://www.ncbi.nlm.nih.gov/bioproject/PRJNA573056
SRR10153128	PRJNA573056	https://www.ncbi.nlm.nih.gov/bioproject/PRJNA573056
SRR10153126	PRJNA573056	https://www.ncbi.nlm.nih.gov/bioproject/PRJNA573056
SRR10153127	PRJNA573056	https://www.ncbi.nlm.nih.gov/bioproject/PRJNA573056
SRR10153133	PRJNA573056	https://www.ncbi.nlm.nih.gov/bioproject/PRJNA573056
SRR10153134	PRJNA573056	https://www.ncbi.nlm.nih.gov/bioproject/PRJNA573056
SRR10153132	PRJNA573056	https://www.ncbi.nlm.nih.gov/bioproject/PRJNA573056
SRR10153129	PRJNA573056	https://www.ncbi.nlm.nih.gov/bioproject/PRJNA573056
SRR10153130	PRJNA573056	https://www.ncbi.nlm.nih.gov/bioproject/PRJNA573056
SRR10153131	PRJNA573056	https://www.ncbi.nlm.nih.gov/bioproject/PRJNA573056

References

1. Achakkagari SR*, et al.* (2022) Genome sequencing of adapted diploid potato clones. *Frontiers in Plant Science* 13.
2. Li Y*, et al.* (2018) Genomic Analyses Yield Markers for Identifying Agronomically Important Genes in Potato. *Molecular Plant* 11(3):473-484.
3. Kyriakidou M, Anglin NL, Ellis D, Tai HH, & Strömvik MV (2020) Genome assembly of six polyploid potato genomes. *Scientific Data* 7(1):88.
4. Kyriakidou M (2020) *Genome assembly and discovery of structural variation in cultivated potato taxa* (McGill University (Canada)).
5. Hoopes G*, et al.* (2022) Phased, chromosome-scale genome assemblies of tetraploid potato reveal a complex genome, transcriptome, and predicted proteome landscape underpinning genetic diversity. *Molecular Plant* 15(3):520-536.
6. Bozan I (2021) Genome analysis of the diploid wild potato Solanum bukasovii. Master of Science (McGill University, https://escholarship.mcgill.ca/concern/theses/fn1073736).
7. Aversano R*, et al.* (2015) The Solanum commersonii Genome Sequence Provides Insights into Adaptation to Stress Conditions and Genome Evolution of Wild Potato Relatives. *The Plant Cell* 27(4):954-968.
8. Pham GM*, et al.* (2020) Construction of a chromosome-scale long-read reference genome assembly for potato. *GigaScience* 9(9).
9. Leisner CP*, et al.* (2018) Genome sequence of M6, a diploid inbred clone of the high-glycoalkaloid-producing tuber-bearing potato species Solanum chacoense, reveals residual heterozygosity. *The Plant journal : for cell and molecular biology* 94(3):562-570.
10. Hardigan MA*, et al.* (2017) Genome diversity of tuber-bearing Solanum uncovers complex evolutionary history and targets of domestication in the cultivated potato. *Proceedings of the National Academy of Sciences* 114(46):E9999.
11. Zhou Q*, et al.* (2020) Haplotype-resolved genome analyses of a heterozygous diploid potato. *Nature Genetics* 52(10):1018-1023.
12. van Lieshout N*, et al.* (2020) Solyntus, the New Highly Contiguous Reference Genome for Potato (Solanum tuberosum). *G3 Genes|Genomes|Genetics* 10(10):3489-3495.
13. Esposito S, Aversano R, Tripodi P, & Carputo D (2021) Whole-Genome Doubling Affects Pre-miRNA Expression in Plants. *Plants* 10(5):1004.
14. Zhang C*, et al.* (2021) Genome design of hybrid potato. *Cell* 184(15):3873-3883.e3812.
15. Zhao K*, et al.* (2022) Incomplete genome doubling enables to consistently enhance plant growth for maximum biomass production by altering multiple transcript co-expression networks in potato. *Theoretical and Applied Genetics* 135(2):461-472.
16. Liao H*, et al.* (2021) High-Throughput MicroRNA and mRNA Sequencing Reveals that MicroRNAs may be Involved in Peroxidase-Mediated Cold Tolerance in Potato. *Plant Molecular Biology Reporter* 39(3):577-594.
17. Kumar A, Kondhare KR, Vetal PV, & Banerjee AK (2019) PcG Proteins MSI1 and BMI1 Function Upstream of miR156 to Regulate Aerial Tuber Formation in Potato1 [OPEN]. *Plant Physiology* 182(1):185-203.
18. Fofana B*, et al.* (2020) Comparative transcriptome expression analysis in susceptible and resistant potato (Solanum tuberosum) cultivars to common scab (Streptomyces scabies) revealed immune priming responses in the incompatible interaction. *PLOS ONE* 15(7):e0235018.
19. Pham GM*, et al.* (2017) Extensive genome heterogeneity leads to preferential allele expression and copy number-dependent expression in cultivated potato. *The Plant Journal* 92(4):624-637.

20. Liu Y (2015) The plant ovule omics: an integrative approach for pollen− pistil interactions and pollen tube guidance studies in solanaceous species. PhD (Université de Montréal, https://doi.org/1866/13589).
21. Yan L*, et al.* (2018) Co-Expression Network-based Analysis associated with potato initial resistance. (bioRxiv).
22. PGSC (2011) Genome sequence and analysis of the tuber crop potato. *Nature* 475(7355):189-195.
